# Recent progress in metal–organic framework/graphene-derived materials for energy storage and conversion: design, preparation, and application

**DOI:** 10.1039/d1sc00095k

**Published:** 2021-03-22

**Authors:** Kaixi Wang, Kwun Nam Hui, Kwan San Hui, Shaojun Peng, Yuxi Xu

**Affiliations:** School of Engineering, Westlake University Hangzhou 310024 Zhejiang Province China xuyuxi@westlake.edu.cn; Joint Key Laboratory of the Ministry of Education, Institute of Applied Physics and Materials Engineering, University of Macau, Avenida da Universidade Taipa Macau SAR China; Zhuhai Institute of Translational Medicine, Zhuhai People's Hospital, Zhuhai Hospital Affiliated with Jinan University Zhuhai Guangdong 519000 China henry2008_ok@126.com; Engineering, Faculty of Science, University of East Anglia Norwich NR4 7TJ UK

## Abstract

Graphene or chemically modified graphene, because of its high specific surface area and abundant functional groups, provides an ideal template for the controllable growth of metal–organic framework (MOF) particles. The nanocomposite assembled from graphene and MOFs can effectively overcome the limitations of low stability and poor conductivity of MOFs, greatly widening their application in the field of electrochemistry. Furthermore, it can also be utilized as a versatile precursor due to the tunable structure and composition for various derivatives with sophisticated structures, showing their unique advantages and great potential in many applications, especially energy storage and conversion. Therefore, the related studies have been becoming a hot research topic and have achieved great progress. This review summarizes comprehensively the latest methods of synthesizing MOFs/graphene and their derivatives, and their application in energy storage and conversion with a detailed analysis of the structure–property relationship. Additionally, the current challenges and opportunities in this field will be discussed with an outlook also provided.

## Abstract 2

This review summarizes comprehensively the latest methods of synthesizing MOFs/graphene and their derivatives, and their application in energy storage and conversion with a detailed analysis of the structure–property relationship.

## Graphical abstract

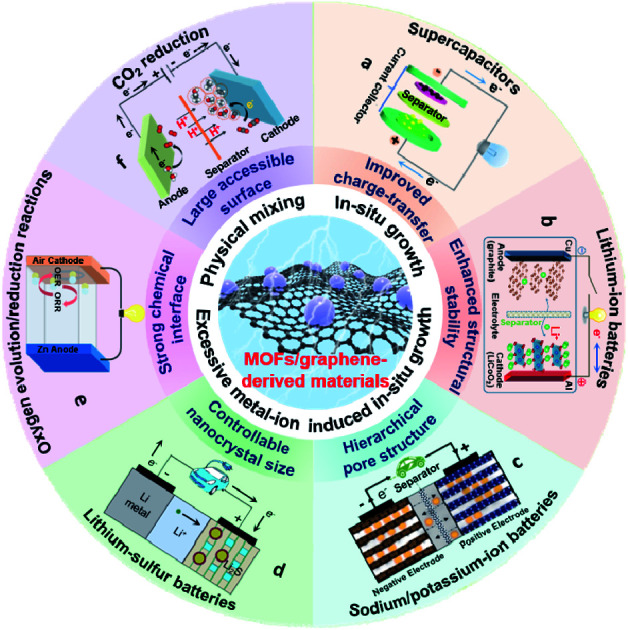

## Introduction

Metal–organic frameworks (MOFs), also known as porous coordination polymers with tunable pore sizes and structures, as well as high specific surface area and pore volume, have been extensively investigated as a new class of inorganic–organic hybrid materials and grown to be an ideal platform for various advanced functional materials and applications. Owing to the easily tunable chemical compositions of the organic linkers and metal nodes (metal ions/clusters), more than 20 000 different MOFs have been designed and synthesized in the past two decades. Their unique structures and properties make them potential candidates in many applications, such as environmental protection,^1–3^ drug delivery,^4,5^ gas adsorption and separation,^6–9^ sensors,^10–12^ catalysis,^13–15^ electrochemical energy storage,^16–21^*etc*. Nevertheless, it should be noted that most pristine MOFs still suffer from the intrinsic drawbacks of low structural stability and poor electrical conductivity, which hinder their practical applications, especially in the field of electrochemical energy storage and conversion.^22,23^ Encouragingly, many efforts have been made to address these issues. Typically, three strategies have been developed, including designing conductive MOFs by using metal ions and organic ligands with loosely bound electrons,^24^ post-synthetic modification of MOFs by modifying the linker and/or metal node, and adsorption/exchange of guest species,^25,26^ and constructing composites with MOFs and other materials,^17,27^ such as MOF/carbon hybrid materials, including MOF/carbon paper,^28^ and MOF/carbon nanotubes.^29^ Among them, the resultant MOF composites have become more prominent due to the synergic effects between the two components.

Graphene, a fascinating two-dimensional (2D) carbon nanosheet with a conjugated hexagonal lattice, has drawn great interest in energy storage and conversion fields due to its huge theoretical surface area, superior electrical conductivity, excellent electrochemical stability, and other unique physical and chemical properties.^30–34^ However, the aggregation and restacking phenomena caused by the strong π–π interaction between graphene layers lead to a great loss of both accessible specific surface area and outstanding single-layer electric properties of graphene, presenting a relatively low electrochemical performance.^35–37^ Assembling graphene and other electrochemical materials to generate a composite has so far been well-accepted to be an effective approach to solve the problem since the introduced component between individual graphene layers can separate them. This composite material is likely to inherit the advantages of the two parent components, but at the same time eliminate their respective shortcomings, which is conducive to the improvement of overall electrochemical performance.

Based on the above analysis, it is desirable to design MOF/graphene-based composites for enhanced electrochemical application. Graphene or chemically modified graphene, because of its high specific surface area and abundant functional groups, provides an ideal template for the controllable growth of MOF particles. With the assembly of MOFs and graphene, the disadvantages of each component can be circumvented and the advantages imparted. (i) The ultrathin graphene nanosheets are stabilized in the presence of MOFs, which ensures that a large accessible surface and many active sites are exposed. (ii) The limitation of poor conductivity of MOFs is alleviated when coupled with graphene, hence promoting electron transport in the whole electrode. (iii) Wrapping MOFs within graphene endows them with much better structural stability during the rapid electrochemical process, resulting in longer cycle life of electrodes.^38^ (iv) The hierarchical pore structures generated in the composite offer a perfect space for accessing the electrolyte and reducing the mass transfer resistance, thus boosting the reaction kinetics.^36,38^ In particular, some unexpected effects take place during the preparation and application process of MOF/graphene-based composites. Ultra-small MOFs with an average size below 10 nm grown on graphene oxide (GO) have been achieved as the excess metal ions facilitate the effective deposition of MOFs by electrostatic and coordination interactions and inhibit their overgrowth.^39^ Moreover, the strong chemical interface between MOF nanocrystals and reduced graphene oxide (rGO) can substantially enhance the adsorption energy and ion transport kinetics for outstanding energy storage performance.^40^ What's more, construction of MOF/graphene nanocomposites with their tunable composition and structures can be easily transformed into single atoms, carbonaceous nanomaterials, metal oxides, sulfides, phosphides, carbides, and nitrides with sophisticated structures,^39,41–47^ showing their unique advantages and great potential in energy storage and conversion, such as supercapacitors,^48^ lithium-ion batteries (LIBs),^49^ lithium–sulfur batteries,^50–53^ sodium-ion batteries, potassium-ion batteries, lithium–oxygen batteries,^54^ CO_2_ reduction,^17,55^ oxygen evolution and reduction reactions,^46,56,57^*etc*.^58,59^

**Fig. 1:**
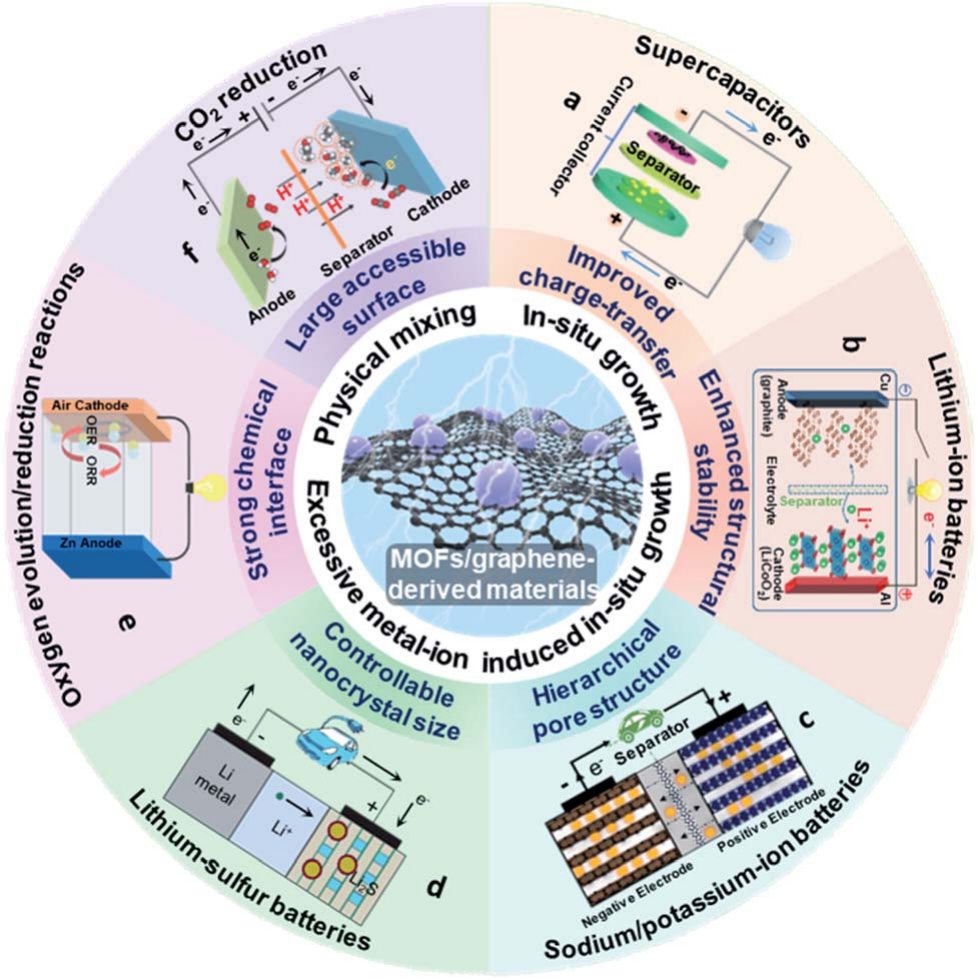
Schematic illustration of the structure, synthesis, properties and energy storage and conversion applications for MOF/graphene-derived materials. (a) Reproduced with permission from ref. 191. Copyright 2020, Elsevier. (b) Reproduced with permission from ref. 197. Copyright 2013, American Chemical Society. (c) Reproduced with permission from ref. 214. Copyright 2018, Wiley-VCH. (d) Reproduced with permission from ref. 221. Copyright 2012, Nature. (e) Reproduced with permission from ref. 152. Copyright 2020, American Chemical Society. (f) Reproduced with permission from ref. 247. Copyright 2018, Wiley-VCH.

Owing to the aforementioned advantages, MOF/graphene-based materials have received enormous attention. The related studies have been increasing in recent years and becoming a hot research topic. Our group have also achieved progress in this research field and thus have a deep understanding of the current research status and challenges.^38–42,44,45,60–64^ Although there are a few reviews about MOF/graphene-based materials and their applications, they do not include the most recent/latest developments of MOF/graphene-based materials in energy-focused applications, or only focus on limited or other aspects (application in environmental remediation, or catalysis).^2,23,43,65^ Different from previous reviews, we will comprehensively summarize the recent advances of MOFs/graphene and their derivatives, and focus on their application in energy storage and conversion (Fig. 1). First, the latest methods of synthesizing MOFs/graphene and their derivatives will be highlighted. Then, the structure–property relationship and its application in energy storage and conversion will be further elucidated in detail. Finally, the challenges and opportunities in this field will be discussed with an outlook also given. We believe this review will attract broad interest from researchers in the chemistry and materials community and stimulate the further development of MOF/graphene-based functional materials.

### Design and preparation of MOF/graphene nanocomposites

In recent years, significant efforts have been made on developing different synthetic strategies for preparing MOF/graphene nanocomposites with various functional components, structures, morphologies, and applications (Table 1). The synthetic strategies of MOF/graphene materials can be generally divided into three types according to the growth and combination of MOFs with graphene: physical mixing, *in situ* growth, and excess metal-ion induced *in situ* growth. No matter which route is used, graphene oxide (GO) is initially used and then reduced to obtain MOF/graphene nanocomposites. It should be pointed out that “graphene” in this review may also refer to GO, and rGO.

**Table 1:** Summary of the preparation and applications of MOF/graphene nanocomposites

Sample	Synthetic strategy	Assisting method	Structure/morphology	BET surface area (m^2^ g^−1^)	Applications	Ref.
GO/Mo-MOFs	Physical mixing	Stirring	Wrapped	—	Derivative for SCs	62
rGO/HKUST-1	Physical mixing	Ultrasonication	Mesoporous	1241	SCs	63
Cu-MOF/rGO	Physical mixing	Ultrasonication	Embedded	1316.2	Electrochemical nitrite sensors	64
PB/GO	Physical mixing	Ultrasonication	Coated	—	Derivative for SIBs	66
GO-HKUST-1	Physical mixing	Grinding	Intercalated thin film	∼600	Derivative for SCs	67
Ni-CoPB@rGO	Physical mixing	Spray pyrolysis	Wrapped	—	Derivative for SIBs	68
Ni-BTC@GO	Physical mixing	Vacuum filtration	Sandwiched film	—	Derivative for overall water splitting and Zn–air batteries	69
MIL-88-Fe/GO	Physical mixing	Stirring, freeze-drying	Interlocked 3D network	—	Derivative for SIBs	72
NH_2_-MIL-125(Ti) MOFs/rGO	Physical mixing	Ultrasonication	Layered	962	Photocatalytic H_2_ production	74
GO/Fe-MOF	Physical mixing	Stirring, freeze-drying	3D porous aerogel	—	Derivative for SCs	75
Fe-CoPBA@GA	Physical mixing	Stirring, hydrothermal reduction	Encapsulated 3D aerogel	—	Derivative for LIBs	76
ZIF-8@rGO	*In situ* growth	High-temperature reduction self-assembly	3D microsphere	—	Oil–water separation	77
CoNi-MOF/rGO	*In situ* growth	Refluxing	Encapsulated	—	Zn–air batteries	78
CoNi-BTC/rGO	*In situ* growth	Solvothermal	3D interconnected structure	—	Derivative for microwave absorption	79
ZIF-8/GO	*In situ* growth	Stirring and aging	Wrapped	—	Derivative for SIBs	80
ZIF-8@GO	*In situ* growth	Stirring	Embedded	—	Derivative for LIBs and SIBs	83
Co-MOF/rGO	*In situ* growth	Solvothermal	Embedded	—	SCs	85
Co-MOF/rGO	*In situ* growth	Stirring	Covered	—	Derivative for LIBs, SIBs, and the HER	87
CoFe-ZIF@GO	*In situ* growth	Solvothermal	Wrapped	—	Derivative for the ORR and OER	88
ZIF-67/GO	*In situ* growth	Hydrothermal	Wrapped	—	Derivative for the ORR and OER	90
ZIF–GO–ZIF	*In situ* growth	Stirring	Sandwiched	—	Derivative for LIBs and SCs	91
CuBTC@GO	*In situ* growth	Stirring and aging	Coated	1772	Adsorbents for CO_2_ capture	92
rGO/UiO-66-NH_2_	*In situ* growth	Solvothermal	Sandwiched	∼800	Organic photosynthesis	93
Fe-MOFs/rGO	*In situ* growth	Solvothermal	Interconnected	—	Derivative for LIBs	94
GA-UiO-66-NH_2_	*In situ* growth	Hydrothermal	3D embedded	707.8	Heavy-metal ion detection	100
MFN@GA/NF	*In situ* growth	Solvothermal	Coated	77.8	OER	106
PB/GO	Excess metal-ion induced *in situ* growth	Stirring	3D porous aerogel	—	Derivative for LIBs	35
Co-MOF–rGO	Excess metal-ion induced *in situ* growth	Annealing	3D encapsulated aerogel	—	PIBs	36
Ni-FePBA/GO	Excess metal-ion induced *in situ* growth	Stirring	3D porous aerogel	—	Derivative for the OER	40

#### Physical mixing

**Fig. 2:**
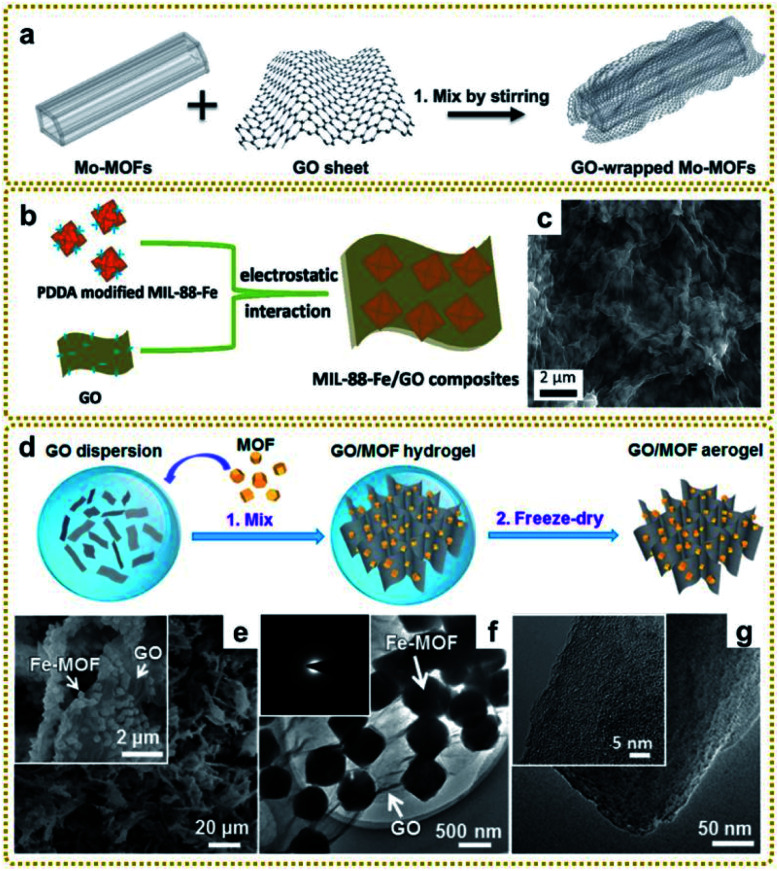
(a) Schematic illustration of the preparation of GO/Mo-MOFs. Reproduced with permission from ref. 66. Copyright 2015, Wiley-VCH. (b) Schematic illustration of the synthesis process of MIL-88-Fe/GO composites. (c) SEM image of MIL-88-Fe/GO composites. Reproduced with permission from ref. 76. Copyright 2018, Royal Society of Chemistry. (d) Schematic illustration of the synthesis process of GO/MOF composite aerogels. (e) SEM images of the GO/Fe-MOF composite aerogel. The inset is a high-magnification SEM image of the GO/Fe-MOF composite aerogel. (f) TEM image of the GO/Fe-MOF composite. The inset is the SAED pattern of the GO/Fe-MOF composite. (g) TEM images of an Fe-MOF crystal. The inset is a high-magnification TEM image of the Fe-MOF crystal. Reproduced with permission from ref. 79. Copyright 2017, American Chemical Society.

Physical mixing is a simple and convenient approach to fabricate MOF/graphene nanocomposites. Firstly, MOFs and graphene are prepared in advance, and then directly mixed with each other to form the composite (Fig. 2a). In this way, a variety of MOF/graphene nanocomposites, including GO/Mo-MOFs,^66^ rGO/HKUST-1,^67^ Cu-MOF/rGO,^68^ ZIF/GO,^69^ PB/GO,^70^*etc*.,^71–74^ were prepared in the early days by stirring, ultrasonication, or grinding the mixtures of MOFs and graphene, as shown in Table 1. However, most of the composites showed poor dispersion due to the weak interaction between MOFs and graphene. To overcome this issue, MOFs are usually modified to enhance the interaction between the mixtures. So far, several efficient modifiers have been reported, such as poly(diallyldimethylammonium chloride) (PDDA)^75,76^ and polydopamine (PDA).^77^ With the decoration of modifiers, the surface of MOFs will be charged. On the other hand, GO is intrinsically negatively charged because of the abundant oxygen-containing groups on the surface. So it is easy for GO to assemble with positively charged MOFs *via* electrostatic interactions. For example, a MIL-88-Fe/GO nanocomposite was fabricated by mixing GO and PDDA modified MIL-88-Fe crystals, which showed tightly interlocked interaction (Fig. 2b and c).^76^ Besides, producing the strong π–π interaction with graphene is also an efficient way to improve the uniformity of MOFs.^78^

Considering the influence of morphology on properties, the preparation of three-dimensional (3D) MOF/graphene nanocomposites has drawn a lot of attention due to their continuous porous network structures, which can provide large accessible surface area, efficient charge and mass transport pathways, and robust mechanical strength. The 3D MOF/graphene aerogel was mainly prepared through a two-step method, involving the self-assembly of MOFs/GO hydrogel and the subsequent reduction process by a reducing agent, hydrothermal reaction, or annealing.^79,80^ Of particular interest, Xu *et al.* prepared various 3D graphene/MOF composites on a large scale and used them as precursors for further application in an all-solid-state flexible supercapacitor, which showed good rate capability with high specific capacitances (Fig. 2d–g).^79^

#### *In situ* growth

**Fig. 3:**
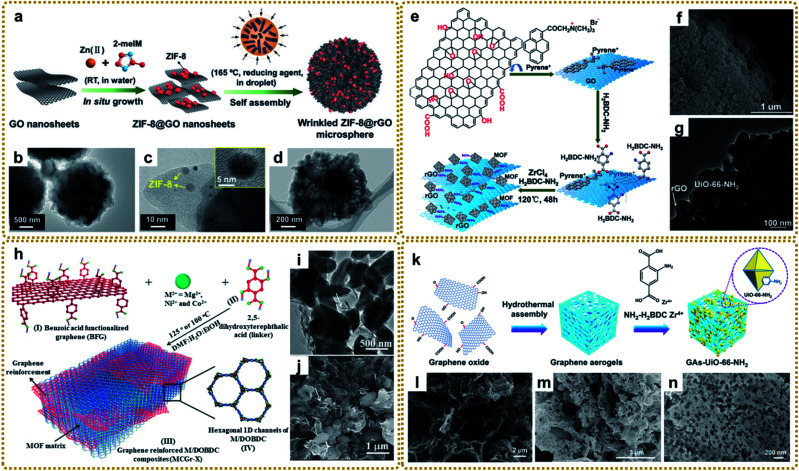
(a) Schematic illustration of the preparation of wrinkled ZIF-8@GO microspheres. (b–d) TEM images of the wrinkled ZIF-8@rGO microspheres: (b) overall view, (c) local view with inset image at high magnification, and (d) plan view. Reproduced with permission from ref. 81. Copyright 2019, Wiley-VCH. (e) Diagrammatic sketch of stepwise fabrication procedures for layered rGO/UiO-66-NH_2_ hybrids. (f) SEM image, and (g) TEM image of MIL-88-Fe/GO composites. Reproduced with permission from ref. 97. Copyright 2015, Royal Society of Chemistry. (h) Schematic representation of the synthesis of covalently linked MCGr-*X* composites. (i) TEM image, and (j) SEM image of NiCGr-10. Reproduced with permission from ref. 100. Copyright 2016, Wiley-VCH. (k) Schematic illustration of the preparation of a GAs-UiO-66-NH_2_ composite. (l) SEM image of GA. (m) SEM image, and (n) TEM image of GAs-UiO-66-NH_2_. Reproduced with permission from ref. 104. Copyright 2019, American Chemical Society.

*In situ* growth is one of the most extensively used strategies for MOF/graphene nanocomposites owing to its facile and fast preparation. Through this way, MOFs grown on graphene will be more uniform, as well as having strong interaction with graphene. In a typical synthesis, GO can coordinate with metal ions first due to its abundant oxygenated functional groups followed by the *in situ* nucleation and growth of MOF nanocrystals when adding the ligands. For instance, wrinkled 3D microspherical ZIF-8@rGO composites were prepared by embedding *in situ* grown ZIF-8 nanoparticles between GO nanosheets, followed by high-temperature reduction self-assembly (Fig. 3a–d). The microspherical composites possess a unique micro/nano hierarchy and show better oil–water separation ability than the individual components.^81^ Moreover, the *in situ* growth strategy has been employed in many other reports (Table 1).^82–94^

The selection of solvents is an important factor in controlling the *in situ* growth rate and structure of MOFs. Due to the difference of ion movement and growth kinetics in different solvents, the sandwiched structure of ZIF–GO–ZIF composites can be synthesized in water instead of the coated structure in methanol, which provides a slower rate of ion depletion.^95^ Shang *et al.* prepared CuBTC@GO composites using a mixed solvent strategy at a low temperature of 323 K for the first time. The authors found that *N*,*N*-dimethylformamide (DMF) promoted the motion of negative anions (BTC^3−^) toward the reaction with the cation (Cu^2+^) and helped the nucleation of CuBTC. Otherwise, CuBTC could not be formed at the relatively low temperature without adding DMF.^96^

Different from the previous synthesis process, the modified GO interacting with ligands prior to metal ions can also affect the structure and therefore properties. Xu *et al.* fabricated a layered rGO/UiO-66-NH_2_ sandwich hybrid using a noncovalent methodology for graphene modification combined with an *in situ* self-assembly technique.^97^ As shown in Fig. 3e, GO was first functionalized with trimethyl-(2-oxo-2-pyren-1-yl-ethyl)-ammonium bromide (pyrene^+^) *via* π–π interaction. Then the attached pyrene^+^ served as an effective mediator to anchor 2-aminoterephthalate acid by electrostatic interaction. Finally, rGO/UiO-66-NH_2_ was obtained after adding Zr^4+^ and a subsequent solvothermal reaction (Fig. 3f and g). The authors highlighted that pyrene^+^ functionalized GO was the key to avoiding its irreversible restacking and aggregation and forming the sandwich-like hybrids, which showed enhanced photocatalytic performance for aromatic chemical synthesis. In this way, many other MOF/graphene composites were prepared.^98,99^ Other studies also talked about the covalent functionalization of GO for MOF/graphene nanocomposites with improved properties.^100–103^ For example, Kumar *et al.* utilized benzoic acid to covalently functionalize the graphene basal plane, then the carboxylate groups of the modified graphene bonded with metal ions (M^2+^), followed by metal ions coordinating with the 2,5-dioxido-1,4-benzene dicarboxylate (DOBDC) linker to form MOFs (Fig. 3h). The as-prepared M/DOBDC-graphene (Fig. 3i and j) presented improved mechanical properties and CO_2_ adsorption characteristics.^100^

Recently, more and more efforts have been devoted to synthesizing 3D MOF/graphene nanocomposites through an *in situ* growth strategy, in which the solution immersion method is the most commonly used and most effective approach. Generally speaking, 3D graphene matrices (*e.g.*, graphene aerogel (GA) and 3D graphene networks (3DGNs)) with various sites for coupling with MOFs were first synthesized by hydrothermal assembly reaction,^104^ chemical vapor deposition (CVD),^105–109^*etc*.^110,111^ Subsequently, the obtained graphene matrix was immersed into the mixed precursor solutions of MOFs for further *in situ* growth as shown in Fig. 3k–n. The enhanced performance of the composites is reasonably attributed to the synergistic interaction between functional MOFs and the 3D graphene matrix with a large surface area and interconnected porous structure.

#### Excess metal-ion induced *in situ* growth

**Fig. 4:**
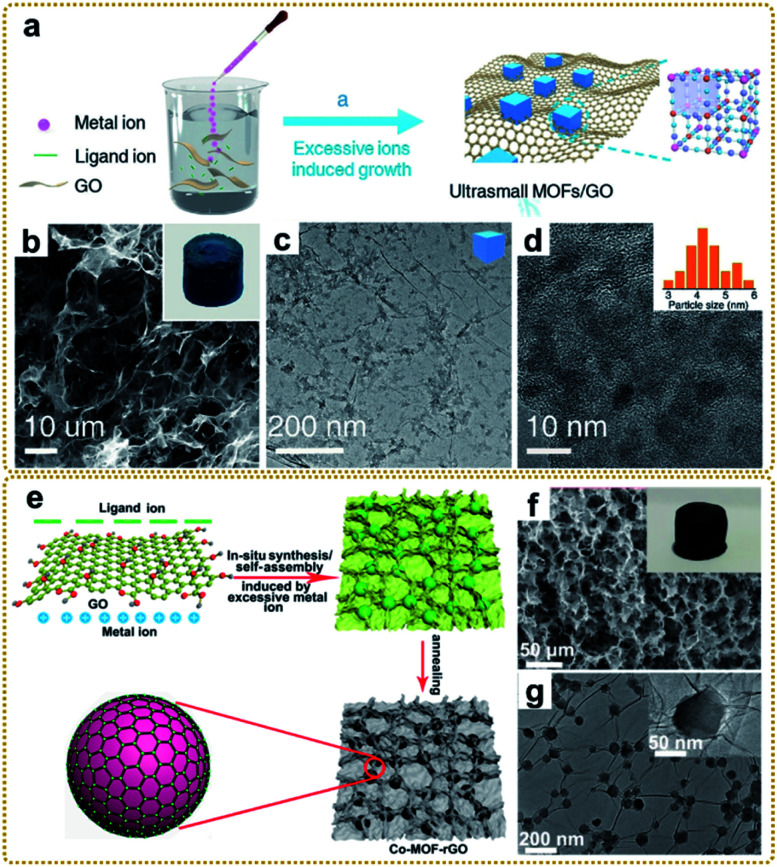
(a) Illustration of the synthesis process of the PB/GO. (b) SEM and (c and d) TEM images of the PB/GO aerogel. The inset of (b) is a photograph of the monolithic PB/GO aerogel. The inset of (d) is a histogram of the size distribution of PB nanoparticles. Reproduced with permission from ref. 39. Copyright 2020, Wiley-VCH. (e) Schematic illustration of the preparation of the Co-MOF–rGO hybrid. (f) SEM image, and (g) TEM images of the Co-MOF–rGO hybrid. The inset of (f) is a photograph of the monolithic Co-MOF–rGO aerogel. Reproduced with permission from ref. 40. Copyright 2020, American Chemical Society.

The traditional *in situ* growth strategy employed to fabricate MOF/graphene nanocomposites usually yields relatively large MOF nanoparticles due to the rapid coordination reaction between metal ions and ligands, which suffer from insufficient available surface area and active sites. To address this issue, our group recently developed a versatile strategy of excess metal-ion induced *in situ* growth to fabricate MOF/graphene nanocomposites with small MOF nanocrystals, through which the obtained MOFs possess an average size below 10 nm.^39^ As show in Fig. 4a, the method has a feasible mechanism: when excess metal ions were added to the homogeneous solution of GO and cyanomethylate ions, some of them reacted with the cyanomethylate anions to form Prussian blue (PB)/Prussian blue analogues (PBAs), and other excess metal ions adsorbed on the surface of PB/PBAs, which promoted the effective deposition of PB/PBA nanoparticles on GO by electrostatic and coordination interactions and inhibited the overgrowth of PB/PBAs. It should be noted that decreasing the content of metal ions would lead to the unsuccessful combination of PB/PBAs on GO, indicating the necessity of excess metal ions in the synthesis system. The resultant PB nanoparticles have an average size of 5.2 nm, distributed uniformly on the GO surface (Fig. 4b–d).

Moreover, this method was also used to prepare a MOF/graphene nanocomposite with strong interfacial interaction. For example, our group synthesized a Co-MOF–rGO nanocomposite with Co-MOF nanocrystals tightly encapsulated in a 3D graphene network *via* strong chemical coupling interaction (Fig. 4e–i) and used it as an electrode material for potassium ion storage.^40^ DFT simulation and experimental results revealed that the strong chemical interface between Co-MOFs and rGO could substantially enhance the adsorption and diffusion of the potassium ion within the MOF nanocrystals, which was the reason for the dramatic enhancement of electrochemical performance. Other MOF/graphene nanocomposites with sophisticated structures and superior properties have also been synthesized using this method.^38,41,44,45,60,62,63,112^

To date, although a large number of MOF/graphene composites have been synthesized and applied in many fields, the MOF types in these materials are very limited, as a lot of work has focused on some common and pristine MOFs, such as Zn-MOF, Fe-MOF, Co-MOF, Ni-MOF, Cu-MOF, and Zr-MOF. The exploration of other pristine MOFs (*e.g.* Al-MOF, K-MOF, conductive MOFs, *etc*.) and modified MOFs (decoration of organic linkers/metal centers, or introduction of other functional species into the MOF) with graphene may present amazing properties and needs to be further investigated.^43^

### Design and preparation of MOF/graphene-derived carbonaceous materials

MOFs have been considered to be a class of ideal precursors to synthesize nanoporous carbon materials *via* direct carbonization due to their large surface area, porous structure, and high carbon content.^113^ However, the application of these derived carbonaceous materials is greatly restricted because there are still some problems with the direct carbonization of dissociative MOFs. For example, the derived carbon materials inevitably aggregate together at high temperature owing to the high surface energy of MOF nanocrystals, resulting in the loss of many active sites. Besides, MOF-derived carbon materials always suffer from poor electrical conductivity due to the relatively low graphitization degree.^114,115^ In this regard, it is intriguing to explore the integration of MOF-derived carbon with graphene, which is expected to be an effective way to increase both density of active sites and electrical conductivity.^116–118^ Consequently, various MOF/graphene-derived carbonaceous materials have been fabricated and show great potential in the field of energy storage and conversion.^119–121^ Among them, zeolitic imidazolate frameworks (ZIFs)/graphene-derived carbonaceous materials are intensively investigated, mainly due to the large surface area and effective nitrogen doping caused by the methylimidazole (MeIM) ligand, which are beneficial for the improved electrical conductivity, catalytic activity, capacitive performance, *etc*.^61^

**Fig. 5:**
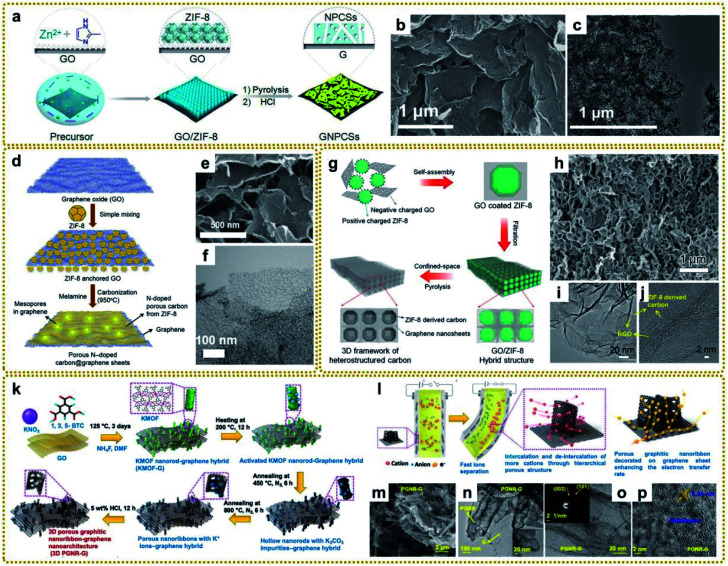
(a) Illustration of the fabrication procedure for GNPCSs. (b) SEM and (c) TEM images of GNPCSs-800. Reproduced with permission from ref. 113. Copyright 2014, Wiley-VCH. (d) Graphical representation of the synthesis procedure of mesoporous-rich nitrogen-doped carbon anchored graphene sheets (MPNC@G) using melamine. (e) SEM and (f) TEM images of MPNC@G-1. Reproduced with permission from ref. 123. Copyright 2019, Elsevier. (g) A schematic illustrating the preparation of a PCF. (h) SEM, (i) TEM, and (j) HRTEM images of a PCF. Reproduced with permission from ref. 121. Copyright 2019, Wiley-VCH. (k) Schematic illustration of the synthesis of a 3D porous graphitic nanoribbon–graphene hybrid (3D PGNR–G) under solvothermal conditions followed by controlled pyrolysis processes. (l) Schematic illustration of the actuation mechanism for remarkable bending actuators based on a 3D PGNR–G/PEDOT:PSS electrode owing to fast ion mobility as well as electron transport through the hierarchical porous structure of the designed 3D PGNR–G in PEDOT:PSS. (m) FESEM image of 3D PGNR–G showing the anchoring of PGNR on wrinkled graphene sheets. (n) TEM image of 3D PGNR–G showing the decoration of PGNR on graphene sheets. The inset is an HRTEM image of 3D PGNR–G. (o and p) HRTEM images of 3D PGNR–G showing the presence of multilayered PGNR and graphene. The inset of (o) is its corresponding SAED pattern. Reproduced with permission from ref. 124. Copyright 2020, Wiley-VCH.

Zhang's group prepared graphene-based nitrogen-doped porous carbon sheets (GNPCSs) upon direct pyrolysis of the 2D sandwich-like GO/ZIF-8 composite and subsequent etching of the possible residual Zn species with acid (Fig. 5a).^113^ To ensure homogeneous and complete growth of ZIF-8 on GO, poly(vinylpyrrolidone) (PVP) was added to modify GO with the amide carbonyl groups, which would coordinate with Zn ions and facilitate the uniform nucleation of ZIF-8. The obtained GNPCSs showed a sheet-like morphology and porous structure (Fig. 5b and c). The synergistic effect between the abundant nitrogen-doped carbon (NC) and continuous graphene conductive network with a well-defined porous structure is crucial for excellent oxygen reduction reaction (ORR) performance. It has been demonstrated that the mesoporous structure can serve as a reservoir for ion storage to facilitate electrolyte transport through shortened diffusion paths, thus enhancing the electrochemical performance.^122^ Nevertheless, ZIF-8-derived NC usually exhibits a microporous structure, which makes electrolyte percolation and ion transport difficult, leading to poor capacity retention at high discharge rates. Han's group reported that melamine could act as a pore-directing additive and expander for a mesoporous-rich carbon hybrid derived from a ZIF-8@GO precursor (Fig. 5d).^123^ The mesopore formation was attributed to the gaseous byproduct evolution of melamine degradation at high temperature for the pore generation and expansion in the carbon architecture. And the mesoporous structure could be optimized by adjusting the content of melamine in the mixed precursor. The obtained melamine-modified samples showed a tiny hollow structure on the graphene sheets (Fig. 5e and f), facilitating efficient electrolyte percolation and ion transport, and bringing about superior Li-ion storage properties.

Although 2D carbon materials derived from MOF/graphene composites have shown unique advantages in many applications and made great progress, currently all of them are difficult to scale due to the time-consuming and careful assembly of MOFs on the surface of graphene. Compared with 2D carbon materials, 3D carbon architectures present faster electron/ion transport and higher ion-accessible surface area. Ding *et al.* developed a facile method to fabricate a 3D porous carbon framework (PCF) constructed from 2D heterostructured carbon nanosheets.^121^ As shown in Fig. 5g, the hybrid GO/ZIF-8 composite was easily prepared by vacuum filtration of a GO coated ZIF-8 composite, which was formed through electrostatic self-assembly of negatively charged GO nanosheets and positively charged ZIF-8 polyhedra. Then a 3D PCF composed of polyhedral-shaped hollow carbon coated with rGO was obtained after confined pyrolysis of a GO/ZIF-8 composite at 900 °C under a nitrogen (N_2_) atmosphere (Fig. 5h–j). The size of the polyhedral macropores can also be adjusted from the nanometer scale to the micrometer scale by employing ZIF-8 polyhedra with different particle sizes. The resultant PCF used as the host material for Li–S batteries exhibited a high discharge capacity and low capacity decay during the cycling test, which stems from their artful design and structure. In another study, Kotal *et al.* reported a scalable synthetic strategy for the formation of 3D porous graphitic nanoribbons anchored on graphene sheets (PGNR–G) with controllable morphology (Fig. 5k).^124^ The formation mechanism was that the K_2_CO_3_ impurities that were generated during the carbonization of a rod-shaped potassium-based MOF (K-MOF) on GO intercalated into the layers of partially decomposed hollow rods, which facilitated the formation of porous nanoribbons. As shown in Fig. 5l, such 3D PGNR–G structure with unzipped layered frameworks favoured faster ion intercalation/deintercalation and electron transport in the electrode. Fig. 5m–p show the 3D nanoarchitecture that unzipped PGNR anchored on wrinkled graphene sheets. When used as an ionic actuator, 3D PGNR–G exhibited breakthrough actuation performance owing to the synergistic effects of PGNR and graphene.

### Design and preparation of MOF/graphene-derived single atom nanocomposites

Single atom materials with isolated metal atoms anchored on supports are emerging as a new frontier in energy conversion applications. The extraordinary characteristics, such as maximum atom-utilization efficiency, special quantum size effect, unsaturated coordination configuration, unique electronic structure, and strong interaction with support materials, endow them with remarkable catalytic activity, selectivity and stability.^125–127^ However, during the synthesis and application of single atoms, they tend to agglomerate to form clusters due to their high surface energy, leading to a decrease in catalytic performance. Using MOF/graphene materials as precursors to prepare single atom nanocomposites is an effective way to overcome the challenge. On the one hand, MOFs with a regular arrangement of metal-based nodes and organic linkers are perfect templates to achieve uniformly dispersed metal single atoms. On the other hand, graphene enables a good conductivity of the derived composites. What's more, graphene is an ideal support for fabricating 2D MOF/graphene derivatives, which is beneficial for more exposed single atoms anchored on the surface, ameliorating the troubling issue that abundant single atoms are buried in MOF-derived solid carbon. To this end, several MOF/graphene precursors have been employed to prepare such satisfactory single atom nanocomposites.

**Fig. 6:**
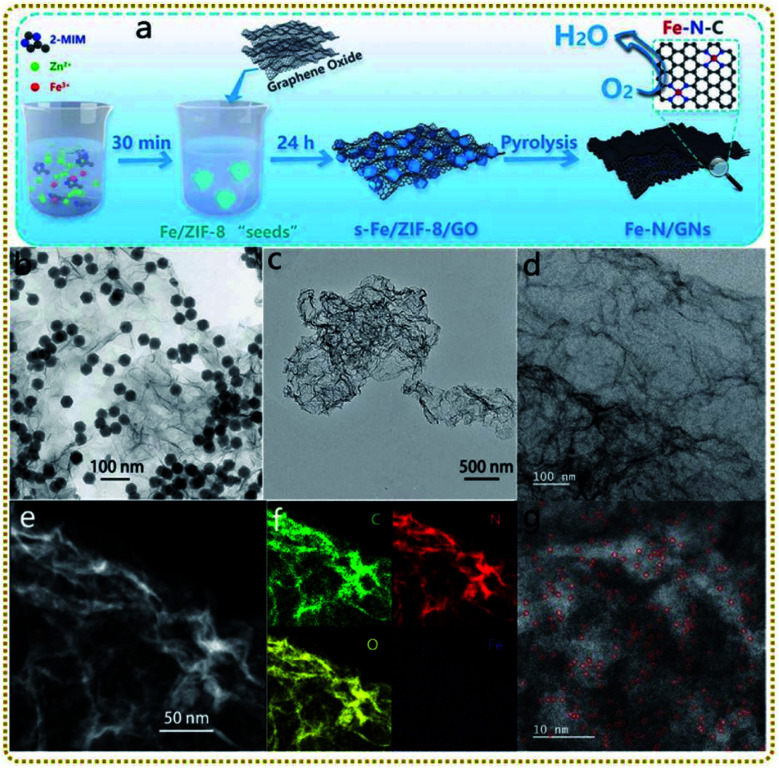
(a) Schematic illustration of the synthesis process of the Fe–N/GNs. (b) TEM image of the s-Fe/ZIF-8/GO. (c) TEM, (d) HRTEM, and (e) STEM images of the Fe–N/GNs. (f) EDS elemental mapping photos in the selected area of (e). (g) HAADF-STEM image of the Fe–N/GNs. Reproduced with permission from ref. 128. Copyright 2020, Wiley-VCH.

Liu *et al.* prepared 2D porous Fe–N-doped graphene nanosheets (Fe–N/GNs) *via* a novel ZIF-8 “thermal melting” strategy by direct high-temperature pyrolysis of the Fe modified ZIF-8 “seeds”/graphene oxide (s-Fe/ZIF-8/GO) precursors at 900 °C (Fig. 6a and b).^128^ The “thermal melting” phenomenon was caused by the high surface energy of s-Fe/ZIF-8 on GO, leading to the fusion and reorganization process under heat-treatment. Thus s-Fe/ZIF-8/GO was converted to 2D porous graphene nanosheets containing ultrathin porous carbon layers covered on the graphene nanosheets (Fig. 6c and d). Fig. 6e–g demonstrate that Fe and N were uniformly doped in Fe–N/GNs and abundant Fe isolated atoms coordinated with N atoms (Fe–N_*x*_) were immobilized on the surface. It is worth mentioning that the Zn signal disappeared in the Fe–N/GNs, mainly because the ultrathin porous carbon reduced the diffusion distance of zinc atoms, making their evaporation easier at high temperatures. As a result, the Fe–N/GN electrocatalyst displayed efficient and stable ORR performances due to the following reasons: (i) single atom Fe active sites anchored on the surface of porous carbon were exposed completely to electrolytes, realizing their fullest utilization; (ii) the high specific surface area of the well-designed structure provided sufficient space to host a high concentration of atomic Fe–N_*x*_ sites; and (iii) the porous graphene ensured high electrical conductivity for fast electron transfer.

Yang *et al.* also synthesized a highly dispersed Fe–N_*x*_ catalyst derived from a graphene supported Fe–Zn-ZIF nanocomposite.^129^ The authors emphasized that the introduction of graphene and PVP was helpful for regulating the size and morphology of ZIFs intercalated into the graphene sheets and avoiding iron particle agglomeration during pyrolysis. They obtained a high Fe–N_*x*_ active site content of 4.29%, surpassing that of most monodisperse non-precious metal catalysts reported. The as-prepared catalyst exhibited a respectable ORR performance with comparable onset and half-wave potentials in acidic medium. Up to now, significant progress has been made toward the fabrication of MOF/graphene-derived single atom nanocomposites with fascinating structures. However, the related research is still in its infancy, and needs to be further conducted.

### Design and preparation of MOF/graphene-derived metal oxides, sulfides, phosphides, carbides, and nitrides with sophisticated structures

Although MOF/graphene composites have achieved great success in the field of energy storage and conversion, they still suffer from some drawbacks, such as low conductivity (MOFs/GO), single pore structure, limited active sites and activity, and absence of multifunctionality, which prevents them from achieving high electrochemical performance and restricts widespread applications.^17,23^ In recent years, metal compounds (oxides, sulfides, phosphides, carbides, and nitrides) have attracted extensive interest due to their potential applications as electrode materials for supercapacitors, lithium-ion batteries, sodium/potassium-ion batteries, lithium–sulfur batteries, oxygen evolution and reduction reactions, CO_2_ reduction, and so on.^18,43^ Encouragingly, these materials can be easily prepared by utilizing a MOF/graphene composite as a precursor. Since the spatially controlled metal node is embedded in the organic ligand environment of MOFs, the prepared metal compounds are usually encapsulated in carbon materials derived from organic ligands, which can result in superior performance. To date, significant progress has been made in the synthesis of lots of MOF/graphene-derived metal compounds with sophisticated structures.

#### Design and preparation of MOF/graphene-derived metal oxides

**Fig. 7:**
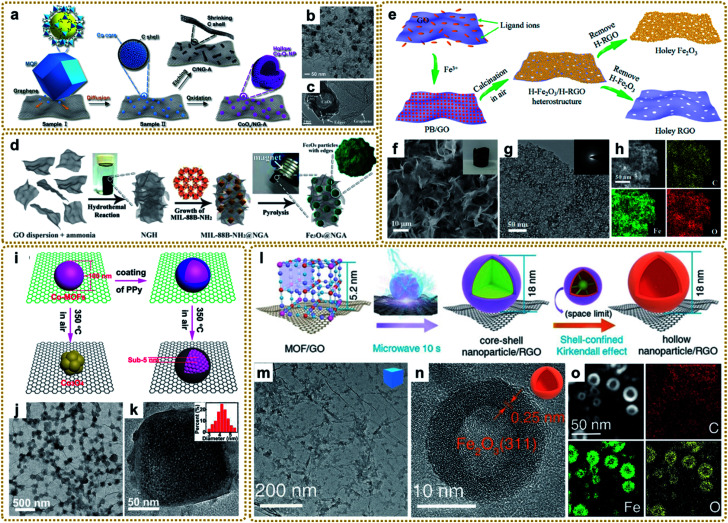
(a) Schematic of the formation process of CoO_*x*_/NG–A and C/NG–A. (b) TEM image of CoO_*x*_/NG–A. (c) HRTEM image of the Co_3_O_4_ phase in CoO_*x*_. Reproduced with permission from ref. 132. Copyright 2017, American Chemical Society. (d) Synthesis process for the Fe_3_O_4_@NGA catalyst. Reproduced with permission from ref. 137. Copyright 2017, Wiley-VCH. (e) Schematic illustration of the synthesis process of the H-Fe_2_O_3_/H-RGO heterostructure. (f) SEM and (g) TEM images, and (h) STEM image and the corresponding elemental mapping images of the H-Fe_2_O_3_/H-RGO. The inset in panel (f) shows the photograph of a monolithic H-Fe_2_O_3_/H-RGO aerogel. The inset in panel (g) shows the SAED pattern of the H-Fe_2_O_3_/H-RGO. Reproduced with permission from ref. 41. Copyright 2018, American Chemical Society. (i) Schematic illustration of the spatially confined pulverization process. (j) TEM image, and (k) HRTEM image of GCP350. The inset of (k) is a histogram of the size distribution of nanocrystals. Reproduced with permission from ref. 42. Copyright 2018, American Chemical Society. (l) Illustration of the synthesis process of hollow nanoparticles/RGO. (m) TEM image of the PB/GO aerogel, (n) HRTEM and (o) STEM images, and elemental mapping images (C, Fe, and O) of S-H-Fe_2_O_3_/RGO. Reproduced with permission from ref. 39. Copyright 2020, Wiley-VCH.

MOF/graphene-derived metal oxides can be obtained by two-step thermal treatment of the precursors.^66,79,105,130–133^ For instance, an rGO/Fe_2_O_3_ composite aerogel was fabricated by annealing a GO/Fe-MOF aerogel under a N_2_ atmosphere at 450 °C, followed by another thermal treatment in air at 380 °C.^79^ Men *et al.* reported that an rGO-wrapped Co@CoO composite with a yolk–shell structure (Co@CoO@rGO) could be achieved by pyrolysis of GO-wrapped ZIF-67 (ZIF-67@GO) in N_2_ with 5 vol% H_2_ at 750 °C. Then, by subsequent pyrolysis of Co@CoO@rGO in air at 350 °C, they obtained the rGO encapsulated hollow Co_3_O_4_ composite (h-Co_3_O_4_@rGO).^133^ Hollow structures can afford large surface areas and shorter diffusion pathways, making it easier to access the electrolyte and reduce the mass transfer resistance, thus boosting the kinetics of electrochemical reactions. Zou's group developed a N-doped graphene aerogel (NG-A) assisted method to prepare monodisperse CoO_*x*_ hollow nanoparticles with highly defective surfaces by a simple thermal activation of bulk Co-MOF crystals (Fig. 7a).^132^ During the first thermal treatment at 750 °C under an argon gas flow, bulk Co-MOFs break into many Co–C core–shell nanoparticles on graphene. Pyrolysis temperature and the assisting graphene were two key factors that influenced the formation of such Co–C core–shell nanoparticles. The sample was then heated at 100 °C in air to obtain CoO_*x*_/NG–A. When exposed to air, the highly active Co cores were oxidized, and the Kirkendall effect occurred, forming hollow cavities due to the different diffusion rates of O atoms in the air and core of Co atoms. The as-obtained hollow CoO_*x*_ displayed abundant edges or corner sites on the surface (Fig. 7b and c), which was helpful for enhanced ORR activity. As a robust Pt-free electrocatalyst for the ORR, the derived CoO_*x*_/NG–A exhibited excellent performance in alkaline electrolyte solution. In addition to the difficult two-step method, the evolved metal oxides can also be prepared by one-step carbonization of the MOF/graphene precursors in inert gas.^94,134–140^ As shown in Fig. 7d, Liang *et al.* synthesized an Fe_3_O_4_ nanoparticles/nitrogen-doped graphene aerogel (Fe_3_O_4_@NGA) hybrid by pyrolyzing MIL-88B-NH_2_@NGA at 900 °C under Ar.^137^ Besides, multi-component bimetal oxides (*e.g.*, CoFe_2_O_4_,^135^ NiCo_2_O_4_/NiO,^136^ and CoO/MoO_2_/CoMoO_4_ (ref. 140)) or metal@metal oxide with core–shell structures^94^ were also prepared in this way.

More conveniently, metal oxides can also be achieved by direct calcination of the MOF/graphene precursors in air.^41,42,62,109,141–143^ For example, our group synthesized a 3DG/Fe_2_O_3_ aerogel with porous Fe_2_O_3_ nanoframeworks wrapped within a graphene skeleton by employing a 3DG/PB aerogel as a template and thermal treatment at 250 °C under air conditions. The hierarchical structure provided a highly interpenetrated porous conductive network as well as abundant stress buffer nanospace for effective charge transport and robust structural stability during electrochemical processes.^62^ To further improve the electrochemical performance, we deliberately designed a double-holey-heterostructure framework with holey Fe_2_O_3_ nanosheets (H-Fe_2_O_3_) tightly and conformably grown on holey reduced graphene oxide (H-RGO) *via* a facile calcination route by utilizing PB/GO composite aerogels as the precursor (Fig. 7e–h).^41^ During the annealing process in air, the transformed Fe_2_O_3_ nanoparticles were interconnected and fused to form a continuous holey nanosheet with abundant nanovoids due to the decomposition of the uniform and dense distribution of PB nanoparticles on GO. Such unique double-holey-heterostructure could accelerate electron and ion transport in unimpeded pathways and promote full utilization of active sites, resulting in enhanced electrochemical performance. We also reported that a coating layer is vital for the transformation of MOF/graphene composites. As shown in Fig. 7i, without the coating of polypyrrole (PPy), Co-MOF particles would transform into metal oxide at 350 °C in air. However, when coated by PPy, Co-MOFs under the same conditions would be *in situ* pulverized into ultrasmall MOF nanocrystals due to the protection of compact PPy layers, which could significantly increase the decomposition temperature and maintain the component stability of Co-MOFs (Fig. 7j and k).^42^ Moreover, our group developed a universal microwave-assisted and confined-diffusion strategy to synthesize a series of hollow nanoparticles, including metal oxides, sulfides, and phosphides, using MOF/GO composites as precursors (Fig. 7l and m).^39^ It can be seen that the obtained hollow Fe_2_O_3_ nanoparticles presented an ultrasmall size and thickness (Fig. 7n and o). The formation mechanism was that graphene that absorbed microwaves quickly created a high-energy environment to decompose PB into uniform core–shell Fe_3_C@C nanoparticles owing to the redeposition of carbon gases generated in this process, then core–shell nanoparticles were further converted into hollow Fe_2_O_3_ through the nano-confined Kirkendall effect of the Fe_3_C core and oxygen by the carbon shell. Notably, the traditional programmed heating strategy could not fabricate such exquisite nanostructure.

#### Design and preparation of MOF/graphene-derived metal sulfides

Metal sulfides, which show more enhanced conductivity than their corresponding oxide counterparts, are recognized as an important class of materials with potential applications in energy storage and conversion. The sulfur atoms with high electronegativity can extract electrons from metal atoms for facilitated electron transport.^144^ Using a MOF/graphene composite as a precursor to synthesize metal sulfides has been extensively studied and various methodologies have been developed till now, including solid-state methods by annealing a mixture of precursor and S powder at high temperatures,^72,76,145–150^ solution-phase methods (*e.g.*, hydrothermal or solvothermal vulcanization reaction) using thioacetamide,^107,151–153^ thiourea,^154^ or Na_2_S^92,155^ as a sulfur source, and gas–solid reaction methods using H_2_S/argon as a reducing gas^156^ or direct carbonization of a S-containing precursor in inert gas.^157^ Thus, many kinds of metal sulfide have been obtained, such as FeS,^60^ FeS_2_,^63,145,146^ Fe_7_S_8_,^76^ NiS,^156^ NiS_2_,^147^ Ni_7_S_6_,^154^ Co_9_S_8_,^150^ Co_9−*x*_Fe_*x*_S_8_,^92^ CoFeS_2_,^148^ ZnSnS_3_,^151^ CuCoS,^152^ CoZnNiS,^155^ and so on.

**Fig. 8:**
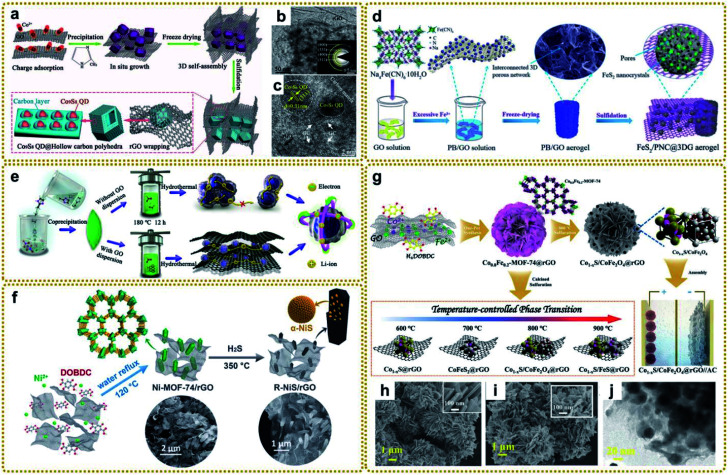
(a) Schematic illustration for the fabrication procedure of (Co_9_S_8_ QD@HCP)@rGO sponge-like composites. (b) TEM and (c) HRTEM images of the (Co_9_S_8_ QD@HCP)@rGO. The inset in (b) is the SAED pattern of the composites. Reproduced with permission from ref. 150. Copyright 2017, Wiley-VCH. (d) Schematic illustration of the preparation process of a multi-scale FeS_2_/PNC@3DG aerogel. Reproduced with permission from ref. 63. Copyright 2019, Royal Society of Chemistry. (e) Schematic illustration of the fabrication process of ZnSnS_3_ and ZnSnS_3_@NG nanocomposites. Reproduced with permission from ref. 151. Copyright 2020, American Chemical Society. (f) Schematic illustration of the synthesis procedure of the R–NiS/rGO from MOF precursors. Reproduced with permission from ref. 156. Copyright 2018, Royal Society of Chemistry. (g) Schematic diagram of the preparation of Co_1−*x*_S/CoFe_2_O_4_@rGO and other temperature-controlled phases. (h) SEM images of Co_0.8_Fe_0.2_-MOF-74@rGO, and (i) SEM and (j) TEM images of Co_1−*x*_S/CoFe_2_O_4_@rGO. Reproduced with permission from ref. 148. Copyright 2020, Wiley-VCH.

As shown in Fig. 8a–c, Chen *et al.* synthesized a fancy composite assembled from a macroporous rGO-wrapped mesoporous hollow carbon polyhedral matrix with Co_9_S_8_ quantum dots embedded in it (denoted as (Co_9_S_8_ QD@HCP)@rGO) using S powder to vulcanize a ZIF-67@GO composite.^150^ The authors found that the presence of coupled rGO not only benefited the growth of small nanoparticles, but also expanded the lattice parameters of Co_9_S_8_ QDs, facilitating sodium storage. Inspired by this work, our group designed a multi-scale nanostructure through one-step sulfidation of PB/GO aerogel in the same way (Fig. 8d).^63^ The derived 3D aerogel, in which ultrafine FeS_2_ nanocrystals were isolated and protected by porous nitrogen-doped carbon nanospheres (PNC) and then encapsulated into 3DG, effectively overcame some key issues of FeS_2_, such as poor electrical conductivity, large volume expansion and agglomeration during electrochemical reactions, and sluggish charge diffusion, showing excellent sodium storage performance. Furthermore, Xie *et al.* prepared an RGO wrapped yolk–shell FeS_2_/C composite *via* facile sulfidation of Fe@RGO, which was evolved by PB@GO in N_2_ at high temperature.^146^ In another study, Xu *et al.* fabricated bimetal ZnSnS_3_ nanodots encapsulated into the interconnected three-dimensional N-doped graphene framework (denoted as ZnSnS_3_@NG) by a hydrothermal vulcanization reaction between a Sn/Zn-bimetal–organic framework and thioacetamide (Fig. 8e).^151^ Owing to the different redox potentials of the two metals, the self-matrix and self-conductivity effect would present simultaneously during the alloying/dealloying process, thus guaranteeing faster charge transfer and effective buffer for the reactive intermediate in lithium storage applications.^158^ In addition, Qu *et al.* prepared a hierarchically porous hybrid electrode material through *in situ* sulfuration of a Ni-MOF-74/rGO architecture in H_2_S/argon mixed gas (Fig. 8f). The as-prepared composite composed of α-NiS nanorods with highly exposed active surfaces decorated on rGO exhibited remarkable supercapacitor performance.^156^

Integrating metal sulfide and metal oxide together to form a hybrid material is an effective way to improve the electrochemical properties because of the realized synergistic effects between good conductivity from the metal sulfide and high redox activity from the metal oxide. Ren *et al.* fabricated an integrated Co_1−*x*_S/CoFe_2_O_4_@rGO nanoflower by *in situ* calcined sulfurization of Co_0.8_Fe_0.2_-MOF-74@rGO at 800 °C, and the temperature-controlled phase transition mechanisms were studied systematically (Fig. 8g–j).^148^ At 600 °C, Co_1−*x*_S was generated preferentially maybe due to the lower bonding energy of Co–S than Fe–S. When the calcination temperature was increased to 700 °C, the high bonding barrier of Fe–S was overcome, but only the CoFeS_2_ phase formed since the activity of oxygen is insufficient to be involved in the reactions at this temperature. As it increased to 800 °C, the metal–sulfur bonds broke and the depleted oxygen components showed high enough activity to produce metal–oxygen bonds, leading to the co-existence of Co_1−*x*_S and CoFe_2_O_4_. However, the high temperature of 900 °C would destroy the Co_1−*x*_S/CoFe_2_O_4_ interfaces and reduce the Fe^3+^ to Fe^2+^ by the carbon species, which further bonded with S to generate stable FeS. Therefore, the appropriate temperature is essential for the formation of the desired sulfurized products.

#### Design and preparation of MOF/graphene-derived metal phosphides

Metal phosphides have had a great impact on energy storage and catalysis related applications due to their high theoretical capacity and activity, low cost, and environmental friendliness.^159,160^ Besides, like sulfur atoms with high electronegativity, phosphorus atoms in metal phosphides can also grip electrons from metal atoms and serve as proton acceptor sites in electrocatalytic reactions.^161^ However, pure metal phosphides face many serious problems during their applications, as can be seen everywhere, which compromises their electrochemical performance. Therefore, it is urgent to develop novel metal phosphide based materials to eliminate these issues. Luckily, it has been demonstrated that deriving metal phosphides from MOF/graphene composites is one of the most effective strategies to significantly improve the electrochemical performance because of the sophisticated structures of the derivatives. Generally, MOF/graphene derived metal phosphides are prepared through a phosphorization process in a tube furnace by using MOF/graphene or the metal/oxide derived from it as a precursor and NaH_2_PO_2_ as a phosphorus source.^91,98,111,112,162–170^ Typically, the precursor and NaH_2_PO_2_ are placed in two separate positions in a porcelain boat with NaH_2_PO_2_ at the upstream side. Then the samples are heated to a certain temperature under an inert gas flow. In this process, PH_3_ begins to be generated with the decomposition of NaH_2_PO_2_ and reacts with the precursor to form a metal phosphide based nanocomposite.

**Fig. 9:**
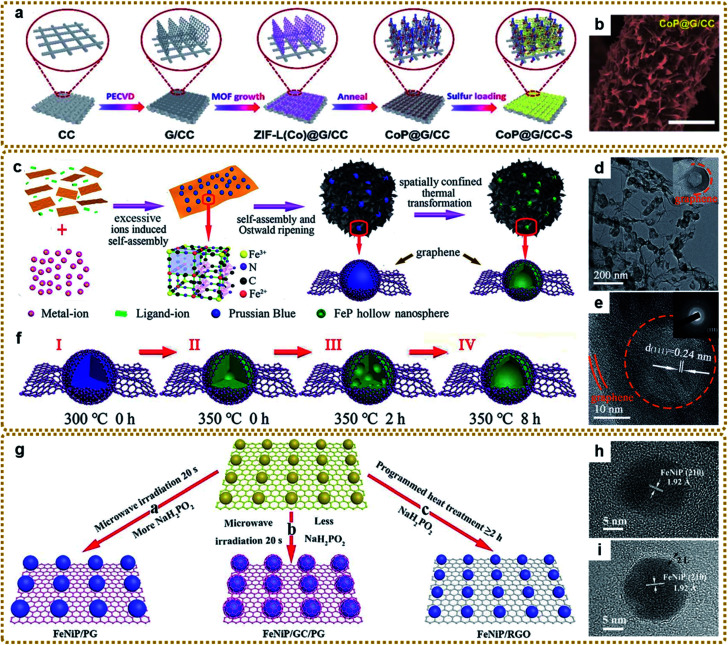
(a) Schematic illustration of the fabrication process of the self-supported CoP@G/CC-S cathode. (b) SEM image of CoP@G/CC hosts. Reproduced with permission from ref. 162. Copyright 2020, Royal Society of Chemistry. (c) Schematic illustration of the fabrication process of the 3DG/FeP composite aerogel. (d) TEM and (e) HRTEM images of 3DG/FeP. The inset in (e) is the SAED pattern of 3DG/FeP. (f) Schematic illustration of the formation process of FeP hollow nanospheres: (I and II) 3DG confined initial decomposition of PB, cyanide ligands decomposed to gas, and the initial phosphating reaction; (II and III) the phase diffused and separated into FeP sub-nanoparticles by the nanoscale Kirkendall effect; (III and IV) the continuing diffusion of the large accumulated phase inside until the formation of the hollow structure. Reproduced with permission from ref. 112. Copyright 2020, Royal Society of Chemistry. (g) Schematic illustration of the formation process of TMP/graphene composites with adjustable heterostructures. (h and i) HRTEM images of FeNiP/PG and FeNiP/GC/PG, respectively. Reproduced with permission from ref. 45. Copyright 2019, Royal Society of Chemistry.

According to this method, Jin *et al.* fabricated a self-supported structure with CoP nanocrystals uniformly anchored on graphene nanoflakes, which vertically grew on conductive carbon cloth (Fig. 9a and b).^162^ When used as a flexible sulfur host for Li–S batteries, the cathode delivered outstanding electrochemical performances owing to the effective immobilization and electrocatalytic interaction of lithium polysulfides with the CoP nanostructures and the synergistic effect of each component. Li *et al.* prepared porous core–shell FeP@CoP microcubes interconnected by RGO using PB as a reactant template. The core–shell structure could offer enough buffer space for volume changes and shorten the Na^+^ diffusion distance for highly efficient sodium storage.^170^ To achieve a larger active surface area and more exposed active sites, Yan *et al.* prepared a Ni_2_P/rGO composite with ultrasmall Ni_2_P nanocrystals (average about 2.6 nm) anchored on rGO *via* one-step low temperature (275 °C) phosphorization of MOF-74-Ni/GO hybrids, which served as a bifunctional catalyst and showed high performance for overall water splitting.^168^ Zhang *et al.* designed hierarchically porous structures composed of FeP hollow nanospheres embedded in a 3DG skeleton (3DG/FeP) through the spatially confined one-step thermal conversion of the 3DG/PB precursor with NaH_2_PO_2_ (Fig. 9c–e).^112^ Such unique structures combined the 3D interconnected conductive network, discrete hollow nanoparticles, and graphene encapsulation effect together, which would promote charge transport and alleviate the volume change, imparting excellent potassium ion storage performance. The formation mechanism of hollow FeP was based on the nanoscale Kirkendall effects of PB shown in Fig. 9f.

To further improve the activity, introduction of another metallic element into the crystal structures of a monometallic phosphide is an effective method to manipulate the electronic state, thereby enhancing the electrochemical properties.^171^ Lu *et al.* synthesized a nanorod-like Fe–Co–P/N-doped graphene hybrid catalyst *via* a dual ligand coordination reaction followed by a phosphorization process. The obtained bimetal phosphide showed remarkably improved water splitting performance compared to its monometallic phosphide counterparts.^167^ Li *et al.* encapsulated hollow (Co,Fe)P nanoframes into a N,P-codoped graphene aerogel for highly efficient water splitting.^172^ In addition, other bimetal phosphide composites (*e.g.*, Fe–Ni–P/rGO^166^ and Ni_*x*_Co_1−*x*_P/rGO^163^) have also been developed to achieve better performance. Different from the traditional method above, our group developed a novel ultrafast microwave-assisted thermal conversion route to synthesize bimetal phosphide/graphene composites. As shown in Fig. 9g–i, FeNiP/P-doped graphene (FeNiP/PG) and core@shell FeNiP@graphitized carbon/P-doped graphene (FeNiP/GC/PG) could be prepared within 20 seconds under microwave irradiation by employing a Ni–Fe PBA/GO/NaH_2_PO_2_ sponge as the precursor.^45^ However, traditional programmed heat treatment only led to FeNiP/non-doped graphene (FeNiP/RGO). A high content of NaH_2_PO_2_ was essential for obtaining FeNiP/PG in this rapid conversion reaction because the decomposition of more NaH_2_PO_2_ absorbed more heat, leading to insufficient temperature for the formation of GC. Furthermore, Na_4_P_2_O_7_ derived from NaH_2_PO_2_ was harmful for the microwave-based thermal transformation reaction.

#### Design and preparation of MOF/graphene-derived metal carbides and nitrides

**Fig. 10:**
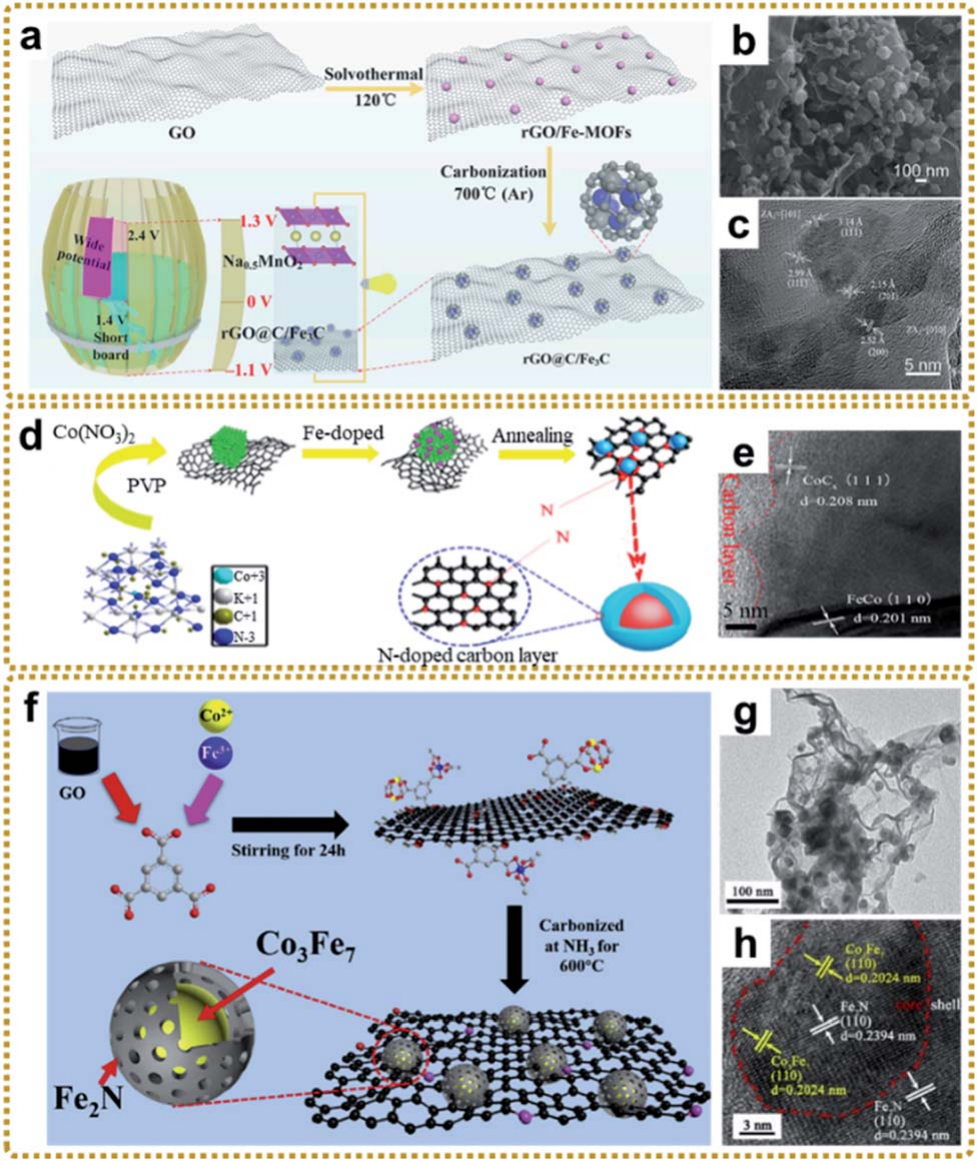
(a) Graphic illustration of the fabrication and application of the rGO@C/Fe_3_C material. (b) SEM and (c) HRTEM images of rGO@C/Fe_3_C. Reproduced with permission from ref. 176. Copyright 2020, Elsevier. (d) Schematic illustration of the synthetic route and structural model of N-doped CoC_*x*_/FeCo@C/rGO, and (e) HRTEM image of N-doped CoC_*x*_/FeCo@C/rGO. Reproduced with permission from ref. 178. Copyright 2019, Elsevier. (f) Illustration of the synthetic process of the Co_3_Fe_7_@Fe_2_N/rGO catalyst. (g) TEM and (h) HRTEM images of Co_3_Fe_7_@Fe_2_N/rGO. Reproduced with permission from ref. 181. Copyright 2020, Elsevier.

Recently, metal carbides and nitrides have garnered much attention because of their high intrinsic conductivity, robust structural stability, abundant catalytic reaction sites, and low cost.^173,174^ Unfortunately, the unmodified pure materials make it difficult to meet the actual needs due to their unfavorable reaction activity. MOF/graphene-derived metal carbides and nitrides can significantly facilitate the reaction kinetics, mainly because the small particles encapsulated in the MOF derived carbon matrix will expose more active sites. What's more, graphene acting as an interconnected matrix offers a highway to promote the electron transfer. In addition, the hierarchical architecture of the composite provides shortened pathways for mass transportation. As mentioned above, the microwave-assisted strategy developed by our group is an effective method to synthesize core–shell metal carbides@C structures.^39^ Besides, most metal carbide derivatives are prepared through one-step carbonization of MOF/graphene precursors in inert gas.^70,175–178^ For instance, Tan *et al.* fabricated an rGO@C/Fe_3_C composite with porous carbon coated Fe_3_C nanoparticles loaded onto rGO nanosheets by *in situ* carbonization of rGO/Fe-MOFs at 700 °C (Fig. 10a–c).^176^ When used as anode materials for battery–supercapacitor hybrid devices, the synergistic effect between Fe_3_C and rGO enhanced the redox reaction of Fe_3_C, resulting in excellent performance. Fang *et al.* designed a nitrogen-doped CoC_*x*_/FeCo@C core–shell structure supported on rGO by a facile thermal treatment of Fe-doped Co_3_[Co(CN)_6_]_2_ MOFs at 800 °C (Fig. 10d and e).^178^ The carbides were formed due to the further reaction of the metal alloy and carbon shell. As a result, the obtained hybrid electrocatalyst showed excellent bifunctional properties for the ORR and OER owing to the multi-component synergistic effect. Besides, Fe/Fe_3_C based materials have also received significant attention due to their durable catalytic activity.^175,177^ However, for metal/metal carbide hybrids, it is difficult to control the reaction degree between the metal and carbon to precisely adjust the ratio of metal to carbide.

In general, MOF/graphene-derived metal nitrides are fabricated *via* ammonolysis of the corresponding precursors by NH_3_ containing gas at high temperature.^179–181^ For example, Zou *et al.* prepared a robust trifunctional electrocatalyst of CoN_*x*_ anchored on a nitrogen-doped graphene aerogel (CoN_*x*_/NGA) by annealing ZIF-67/GA at 500 °C under an ammonia atmosphere.^179^ The CoN_*x*_/NGA hybrid presented exceptional catalytic performance comparable to that of precious metals due to the plentiful dual active sites of CoN_*x*_ and N_*x*_C and hierarchically porous structure. Kwag *et al.* synthesized 2D bimetal Fe_2_Ni_2_N/rGO nanohybrid sheets by nitridation of Ni_2.25_Fe_0.75_[Fe(CN)_6_]_2_/rGO nanosheets in a N_2_/NH_3_ co-atmosphere at 450 °C, which showed outstanding OER performance.^180^ Furthermore, Liang *et al.* constructed core–shell Co_3_Fe_7_@Fe_2_N nanoparticles supported on rGO by direct pyrolysis of FeCo-BTC@GO at 600 °C in NH_3_ (Fig. 10f–h).^181^ The Co_3_Fe_7_ alloy core could promote electron conductivity, and the porous Fe_2_N shell could provide abundant active sites of Fe–N–C apart from the enhanced stability of the material during the catalytic reaction. Thus, such unique structures exhibited superior ORR and OER reactivity. Bimetallic or heterostructure nitrides may provide more favorable electronic configurations than bare nitrides for better electrochemical performance, so further in-depth research into the structure–performance relationship is of great significance.

## The application of MOF/graphene-derived nanocomposites in energy storage and conversion

Recently, pristine MOFs have aroused great interest among researchers for energy storage and conversion due to their remarkably high porosity and surface area, as well as tunable composition and pore structure. In particular, MOFs containing redox-active metal centers are of extraordinary interest for delivering electrochemical activity.^17^ However, the electronically insulating nature of most MOFs becomes a major obstacle to their electrochemical applications. Under this circumstance, developing intrinsically conductive MOFs has been recognized as a promising way to essentially solve this problem.^182^ Conductive MOFs inherit merits beyond traditional MOFs and they can also trigger faster electron transport during electrochemical reactions. Undoubtedly, conductive MOFs have broadened the applications of MOFs in supercapacitors, batteries, electrocatalysis, *etc*.^183^ Nevertheless, it seems difficult for conductive MOFs to possess both high conductivity and high specific surface area simultaneously, which will compromise the electrochemical performance.^184^ For the conductive MOF/graphene composite, graphene can not only serve as an ideal platform for the selective and uniform growth of conductive MOFs, but can also provide continuous pathways to ensure the high electrical conductivity of the entire electrode.^185^ Besides, the presence of graphene may also affect the size and stability of conductive MOFs, like in traditional MOFs. Conductive MOF/graphene-based materials are just in their infancy and more efforts are still required in the future. In this part, we mainly discuss MOF/graphene-derived nanocomposites for energy storage and conversion applications.

### Supercapacitors

Supercapacitors (SCs), also known as electrochemical capacitors, are considered to be one of the most efficient and favored electrochemical energy storage devices, due to their various merits including fast charge–discharge rate, high specific density, long cycle life, and low cost.^186,187^ At present, SCs are widely used in energy/power requiring portable consumer electronics, and industrial devices.^19,61^ Based on the different charge storage mechanisms, SCs can generally be divided into two categories: electric double-layer capacitors (EDLCs) and pseudocapacitors. In EDLCs, electrical energy storage is based on the formation of a double layer of electrolyte ions on the surface of the electrode *via* electrostatic charge adsorption. Carbon materials, such as activated carbon (AC), graphene, and other porous carbons, are typical EDLC electrode materials. They possess a fast charge–discharge rate and long lifetime but low capacitance and energy density. In pseudocapacitors, the energy is stored through rapid and reversible redox reaction with electron transfer on the interface of the electrode and electrolyte. Conducting polymers and metal compounds are commonly used as pseudocapacitive electrode materials, showing large capacitance and delivering high energy density. However, they suffer from relatively poor cycling stability and low power density.^188^ Thus, both EDLCs and pseudocapacitors have their inherent drawbacks, which limits their widespread applications that require high energy density and power density at the same time. It is well known that electrode materials dominate the performance of SCs. Ideal electrode materials for high-performance SCs need to have some vital characteristics, such as large specific surface area, hierarchically porous structure, superior conductivity, and more active sites,^19,189^ which are highly consistent with the properties of materials derived from MOF/graphene nanocomposites. In this regard, developing MOF/graphene-derived nanocomposites with sophisticated structures is an effective strategy for advanced SC applications.

**Fig. 11:**
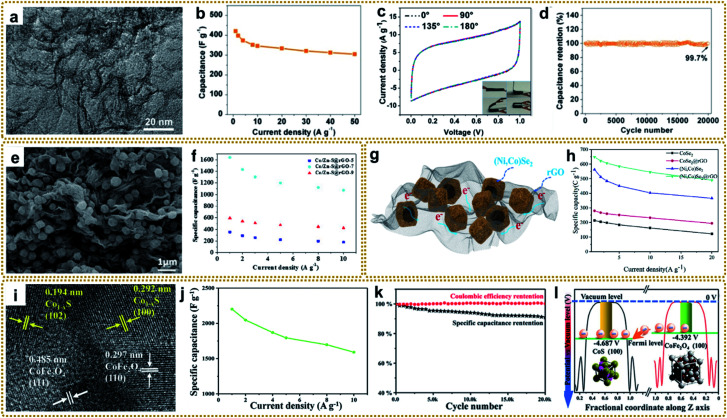
(a) TEM image of C/NG-A. (b) Specific capacitance of C/NG-A. (c) Bending test of C/NG-A SSCs. (d) Cycling stability of C/NG-A SSCs at 20 A g^−1^. Reproduced with permission from ref. 132. Copyright 2017, American Chemical Society. (e) SEM image of Co/Zn–S@rGO. (f) Specific capacitances of the Co/Zn–S@rGO electrode at various current densities. Reproduced with permission from ref. 191. Copyright 2020, Elsevier. (g) Schematic diagram displaying the merits of the (Ni,Co)Se_2_@rGO electrode. (h) Specific capacity calculated from discharge curves and corresponding capacity retention. Reproduced with permission from ref. 193. Copyright 2020, Elsevier. (i) TEM image of Co_1−*x*_S/CoFe_2_O_4_@rGO. (j) The specific capacitance of sample-800 at different current densities. (k) Cycling performance of sample-800 at a current density of 10 A g^−1^. (l) Potentials of CoFe_2_O_4_ and CoS low-index surfaces. Reproduced with permission from ref. 148. Copyright 2020, Wiley-VCH.

Xia *et al.* prepared a nitrogen-rich porous carbon–graphene aerogel electrode (C/NG-A) through carbonization of Co-MOF/NG-A followed by etching in concentrated hydrochloric acid.^132^ As shown in Fig. 11a, C/NG-A was constructed from interconnected graphene networks and porous carbons, which provide a large ion-accessible surface area and efficient ion/electron transport pathways. As a result, the C/NG-A yielded a high capacitance of 421 F g^−1^ at 1 A g^−1^, outperforming most reported nitrogen-doped graphene materials. Furthermore, even at a high current density of 50 A g^−1^, 72.5% of its initial value was still retained, suggesting fast ion and electron transport within C/NG-A at high current density (Fig. 11b). When used in flexible all-solid-state supercapacitors (SSCs), the as-prepared supercapacitor could be bent without capacitance loss, as proved by the CV curves under different bending angles (Fig. 11c). Besides, the C/NG-A based SSCs also presented outstanding cycling stability (Fig. 11d). Finally, the C/NG-A delivered a high power density (500 W kg^−1^) at an energy density of 33.89 W h kg^−1^. To achieve higher capacitance and energy density, various metal compound/graphene composites were designed, such as rGO/Fe_2_O_3_,^79^ rGO/MoO_3_,^66^ rGO/Co_3_O_4_,^95^ 3DGN/Mn_2_O_3_,^108^ R–NiS/rGO,^156^ rGO@C/Fe_3_C,^176^ CoZnNiS@CNTs/rGO,^155^ and so on.^148,190–193^ In particular, Xin *et al.* fabricated a 3D Co/Zn–S@rGO film through a mild *in situ* synthesis method and vulcanization of Co/Zn-MOF@GO film.^191^Fig. 11e shows that Zn_0.76_Co_0.24_S nanoparticles were homogeneously embedded between rGO sheets, and also acted as spacers to prevent the rGO sheets from agglomerating. The resultant 3D sandwich film with porous and continuous networks was employed as a binder-free electrode for SCs and exhibited a high capacitance of 1640 F g^−1^ at a current density of 1 A g^−1^. The assembled symmetric SCs (Co/Zn–S@rGO-7//AC) showed an ultra-high energy density (91.8 W h kg^−1^) at a power density of 800 W kg^−1^ and excellent cycle stability (90.3% capacity retention after 8000 cycles at 10 A g^−1^).

As mentioned earlier, hollow structures can provide large accessible surface areas and shorter diffusion pathways for electrolyte ions, which is beneficial for rapid mass transfer and improved electrochemical performance. Thus, it is very attractive to construct metal compound/graphene composites with hollow structures for high-performance SCs. For example, Liu *et al.* synthesized a hierarchical hollow (Ni,Co)Se_2_ nanocubes@rGO hybrid, in which the porous rGO not only acted as the conductive network to increase conductivity, but also served as a protector and space separator to maintain structural integrity.^193^ Besides, the hollow (Ni,Co)Se_2_ nanocubes could offer abundant electrochemically active sites, as well as enhancing the diffusion of ions in electrolyte (Fig. 11g). The as-prepared (Ni,Co)Se_2_@rGO electrode achieved a high specific capacity of 649.1 C g^−1^ at 1 A g^−1^ (Fig. 11f). The resulting (Ni,Co)Se_2_@rGO//AC hybrid supercapacitor delivered a high energy density of 52.6 W h kg^−1^ at a power density of 803.4 W kg^−1^. In another separate study, Zardkhoshoui and co-authors assembled a hybrid supercapacitor by using a graphene wrapped multi-shelled NiGa_2_O_4_ hollow sphere as a positive electrode material and a graphene-wrapped yolk–shell NiFe_2_O_4_ hollow sphere as a negative electrode material.^190^ Benefiting from their sophisticated structures, the fabricated asymmetric device showed an exceptional energy density of 118.97 W h kg^−1^ at 1702 W kg^−1^ and an excellent robustness of 92.1%.

Interfacial engineering has been demonstrated to be a practicable strategy to improve the electrochemical performance. The modified interface can optimize the electronic environment, facilitate electron and mass transportation, and expose more active sites, which is beneficial for fast electrochemical reaction kinetics.^194^ Recently, an integrated Co_1−*x*_S/CoFe_2_O_4_@rGO nanoflower with abundant Co_1−*x*_S/CoFe_2_O_4_ heterointerfaces was reported for high-performance SC application (Fig. 11i–l).^148^ It yielded an ultrahigh specific capacity of 2202 F g^−1^ at 1 A g^−1^ (Fig. 11j), which was much higher than that of the individual components. In addition, it showed remarkable cycling stability with a capacitance retention of 90% after 20 000 cycles at 10 A g^−1^ (Fig. 11k). Furthermore, the fabricated Co_1−*x*_S/CoFe_2_O_4_@rGO//AC asymmetric supercapacitor achieved an excellent energy density up to 61.5 W h kg^−1^ at 700 W kg^−1^. Density functional theory (DFT) calculation revealed that CoFe_2_O_4_(100) exhibited a higher potential than CoS(110), and the electrons could transfer from the conductivity-poor CoFe_2_O_4_ to conductive CoS through the heterointerfaces and then to rGO, thus realizing rapid Faraday reactions (Fig. 11l).

To sum up, morphology engineering, interface engineering, and doping engineering are effective methods to improve the electrochemical performance. However, it is hard for a single strategy to simultaneously meet the performance requirements of supercapacitors in practical applications, including large capacity, long cycle stability, and high power and energy density. In order to develop more efficient MOF/graphene-based materials, the synergy of combining these strategies may be a promising solution.

### Lithium-ion batteries

In today's society, lithium-ion batteries (LIBs) are indispensable in people's daily lives. Since the commercialization of LIBs in 1991, they quickly governed the battery market owing to their large voltage, high energy density, and long cycle life,^195^ and are widely used in many fields, such as portable electronic devices, hybrid electric vehicles, and power grids.^196,197^ In conventional LIBs, graphite is commonly used as the negative and lithiated transition metal oxides (*e.g.*, LiFePO_4_, LiMn_2_O_4_, and Li[Ni_*x*_Co_*y*_Mn_*z*_]O_2_) as the positive electrode materials.^198,199^ Although great progress has been achieved, they are still insufficient for the above applications due to the relatively low capacity, as well as low energy and power density.^16^ Recently, tremendous efforts have been devoted to fabricating MOF/graphene-derived nanocomposites with sophisticated structures, which are considered to be one of the most promising candidates to improve the LIB performance.

As an attractive anode material in LIBs, graphene shows a favourable low operating potential and long cycle life. However, it still suffers from unsatisfactory electrochemical activity, sluggish Li ion transport rate and insufficient accessible active sites, which leads to low energy and power density.^200,201^ To address these issues, researchers have paid attention to construct a porous structure or introduction of heteroatoms (*e.g.*, N, P, S, and B) to the graphene frameworks. The porous structure can provide a larger surface area and more active sites for interfacial electron transfer reaction. Moreover, the pores can play a role in alleviating the volume change during the charging and discharging process to reduce capacity fading. Meanwhile, the doping of heteroatoms can improve the conductivity and reduce the diffusion resistance of Li ions within graphene frameworks.^202^ For example, nitrogen-doped porous graphene hybrid nanosheets derived from a ZIF-8/GO composite were reported to be a superior anode material for LIBs with a high reversible specific capacity of more than 700 mA h g^−1^.^118,120^ Furthermore, reasonable control of the pore size distribution to the mesoporous range can accelerate the migration of ions, since the micropores will restrict the ion/electrolyte transport at high discharge rates.^203^ Therefore, Gayathri *et al.* prepared mesoporous-rich nitrogen-doped carbon@graphene nanosheets derived from a ZIF-8 precursor using melamine as a pore expanding agent and surface modifier, which exhibited high specific capacity and excellent long cycle life compared to the melamine-unmodified anode.^123^

**Fig. 12:**
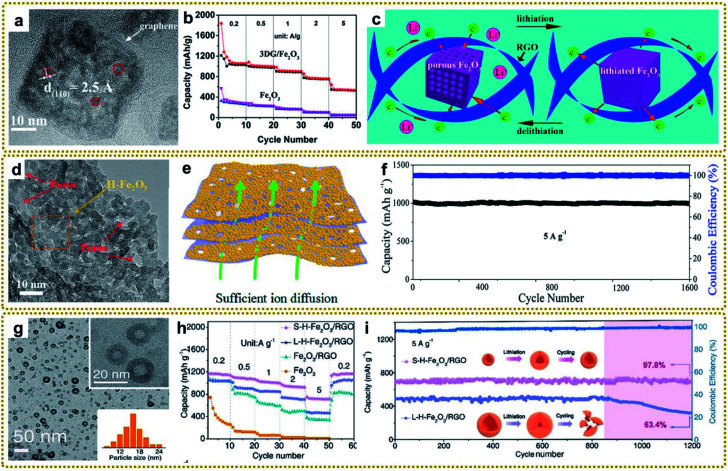
(a) HRTEM image of 3DG/Fe_2_O_3_. (b) Rate performance of 3DG/Fe_2_O_3_ and Fe_2_O_3_, respectively. (c) The schematic of delithiation/lithiation reactions of 3DG/Fe_2_O_3_. Reproduced with permission from ref. 62. Copyright 2017, American Chemical Society. (d) HRTEM image of the H-Fe_2_O_3_/H-RGO with the red arrows indicating the nanopores from the H-RGO beneath the H-Fe_2_O_3_ (yellow arrow). (e) Schematic illustration of the H-Fe_2_O_3_/H-RGO heterostructure. (f) Cycling performance of the H-Fe_2_O_3_/H-RGO anode at 5 A g^−1^ for 1600 cycles. Reproduced with permission from ref. 41. Copyright 2018, American Chemical Society. (g) TEM image of S-H-Fe_2_O_3_/RGO. The insets are the TEM image at high magnification and a histogram of the size distribution of H-Fe_2_O_3_ nanoparticles, respectively. (h) Rate performance of S-H-Fe_2_O_3_/RGO, L-H-Fe_2_O_3_/RGO, Fe_2_O_3_/RGO, and Fe_2_O_3_ anodes. (i) Cycling performance of S-H-Fe_2_O_3_/RGO and L-H-Fe_2_O_3_/RGO at 5 A g^−1^ for 1200 cycles. Reproduced with permission from ref. 39. Copyright 2020, Wiley-VCH.

In contrast to the Li^+^ intercalation storage mechanism of carbon materials, metal compounds based on reversible conversion reactions can provide higher capacity, energy density and power density.^17^ However, metal compounds often exhibit poor conductivity and large volume expansion during the charging and discharging process, which leads to sluggish electron transport and structure pulverization in the lithiation reaction, resulting in rapid deterioration of battery performance. Combining metal compounds with graphene to form a hybrid material can effectively solve these problems because of the excellent conductivity and confinement effect of graphene. Based on these understandings, various metal compounds/graphene have been synthesized, including Fe_2_O_3_/RGO,^39,41,62,75,105^ Fe–Co oxide@GA,^80^ ZnO/C/rGO,^86^ rGO/Co_3_O_4_,^95,130,133^ 3DGN/CuO,^109^ MnO/C@rGO,^134^ 3DG@Co_0.85_Se@C,^204^ N–ZnSe@rGO,^87^ FeP@C/rGO,^98^ ZnSnS_3_@NG,^151^*etc*.^140,143^ Among them, our group deliberately synthesized a 3D graphene/Fe_2_O_3_ aerogel (3DG/Fe_2_O_3_) with porous Fe_2_O_3_ nanoframeworks well encapsulated within a graphene skeleton by employing a 3DG/MOF aerogel as a template (Fig. 12a).^62^ When used as a free-standing anode for LIBs, 3DG/Fe_2_O_3_ exhibited an ultrahigh capacity of 1129 mA h g^−1^ at 0.2 A g^−1^ and excellent cycling stability (Fig. 12b). The superior performance could be generally ascribed to rapid electron/ion transport in the hierarchical structure and integrated structure of porous Fe_2_O_3_ protected by the robust 3DG network during the cycling process (Fig. 12c). To further promote ion transport in the graphene framework, we designed a H-Fe_2_O_3_/H-RGO double-holey-heterostructure framework, in which holey Fe_2_O_3_ nanosheets are intimately grown on holey RGO (Fig. 12d).^41^ The in-plane nanopores could serve as open channels to promote ion and electron transport, as well as allowing for sufficient utilization of active sites throughout the highly compact electrode (Fig. 12e). As a result, the obtained H-Fe_2_O_3_/H-RGO heterostructure anode delivered ultrahigh gravimetric, areal, and volumetric capacities. Besides, it also achieved extraordinary rate performance and cycling stability with a capacity retention of 96.3% after 1600 cycles (Fig. 11f).

Moreover, reducing the dimensions of active materials is an efficient route to enhance the electrochemical performance owing to the short distance for Li^+^ to transport to the reaction sites. Our group developed a facile microwave-assisted and shell-confined Kirkendall diffusion strategy to prepare ultrasmall hollow nanoparticles using ultrasmall MOFs/GO as a self-sacrificial template.^39^ The obtained ultrafine hollow Fe_2_O_3_ nanoparticles were uniformly distributed on the surface of RGO (Fig. 12g). As expected, the ultrasmall hollow Fe_2_O_3_ nanoparticles on RGO (S-H-Fe_2_O_3_/RGO) delivered high capacities and superior rate capability, much better than those of a large hollow Fe_2_O_3_ nanoparticle/RGO composite (L-H-Fe_2_O_3_/RGO) and a solid Fe_2_O_3_ nanoparticle/RGO composite (Fe_2_O_3_/RGO) (Fig. 12h). Furthermore, the S-H-Fe_2_O_3_/RGO presented a highly reversible capacity of 684 mA h g^−1^ with a capacity retention of 97.8% after 1200 cycles. In contrast, the L-H-Fe_2_O_3_/RGO showed obvious capacity decay after 850 cycles and lower capacity retention of 63.4% under the same conditions. This significant difference demonstrated that ultrasmall hollow Fe_2_O_3_ nanoparticles possessed better stress migration ability than the large ones, which broke into small pieces after cycling (Fig. 12i).

In addition to the MOF/graphene-derived nanocomposites discussed above as anode materials for lithium-ion batteries, MOFs have also been explored as cathode materials due to their redox active sites from both transition metal cations and/or organic linkers with redox active functional groups. Besides, the regular channels of MOFs can facilitate Li^+^/Na^+^ diffusion and insertion.^17,205^ For example, Awaga and co-workers reported Cu-AQDC with redox activity on both the metal ions and organic linkers for cathode materials of LIBs. The charge–discharge curves involved two distinct electrochemical processes, that is, two-electron redox reaction from anthraquinone groups in the ligands and one-electron reaction from the Cu^II^/Cu^I^ redox couple. As a result, Cu-AQDC exhibited a high specific capacity of 147 mA h g^−1^.^206^ However, Cu-AQDC showed large capacity fading in 50 cycles, which was mainly caused by the poor electrical conductivity and highly localized electron density. The conductive MOFs may open up a new path for better electrochemical performance because their electrical conductivities and porous structures are favourable for electron and ion transport in the framework.^207^ The potential applications of conductive MOFs in lithium/sodium-ion batteries have been confirmed by Dincă and co-workers who showed that the prepared Ni_3_(HITP)_2_ MOF with high bulk electrical conductivity delivered high performance in electrochemical supercapacitors,^208^ as well as some other researchers using conductive MOFs in various battery systems, such as Na^+^/Zn^2+^ batteries,^209,210^ Li–S batteries,^211^ and so on. Moreover, the emerging new MOFs based on new ligands (*e.g.* organic Li-ion compounds) are promising cathode materials for lithium/sodium-ion batteries.^212^ What's more, we believe that the composites composed of traditional or conductive MOFs with graphene could show surprising performance due to their synergistic effect, which has been demonstrated by our group.^38^

### Sodium/potassium-ion batteries

In recent years, LIBs have expanded their application in the field of electric vehicles and large-scale electrical energy storage systems, and achieved considerable progress. Nevertheless, the relatively scarce lithium resources and high cost greatly hinder the development.^213^ Compared to lithium, sodium and potassium are much more abundant in the crust, and thus cheaper. Besides, sodium and potassium exhibit similar physical and chemical properties to lithium due to the same alkali metal group. Moreover, sodium/potassium-ion batteries have similar electrical storage mechanisms to that of LIBs. Therefore, sodium/potassium-ion batteries have garnered extensive attention and are expected to be potential alternatives to LIBs.^34,214,215^

It has been proven that the commonly used graphite in LIBs is almost electrochemically inactive in sodium-ion batteries (SIBs) due to the energy instability of Na–graphite intercalation compounds.^216,217^ In addition, many electrode materials suffer from sluggish reaction kinetics and inferior electrochemical activity because of the large ionic radius and heavier atomic mass of Na^+^, which leads to low specific capacity and poor cycling stability.^218^ Therefore, it is urgent to develop desirable electrode materials for high performance SIBs. Meanwhile, thanks to the unique advantages mentioned earlier, MOF/graphene-based materials seem to be one of the ideal choices. For instance, our group synthesized a 3DG wrapped PB aerogel, and used it as a free-standing cathode material for SIBs. Excitingly, it delivered an extraordinary rate performance (84 mA h g^−1^ at 25C) and long-term cycling stability (90% capacity retention after 1000 cycles at 10C), which was attributed to the highly efficient electron and ion transport in the whole electrode.^38^ Furthermore, MOF/graphene-derived nanocomposites, especially sulfides,^60,63,70,72,76,145,150^ selenides,^84,87^ and phosphides,^91,111,170^ are widely studied for SIBs, due to their environmental benignity and higher theoretical specific capacities based on conversion reactions.

**Fig. 13:**
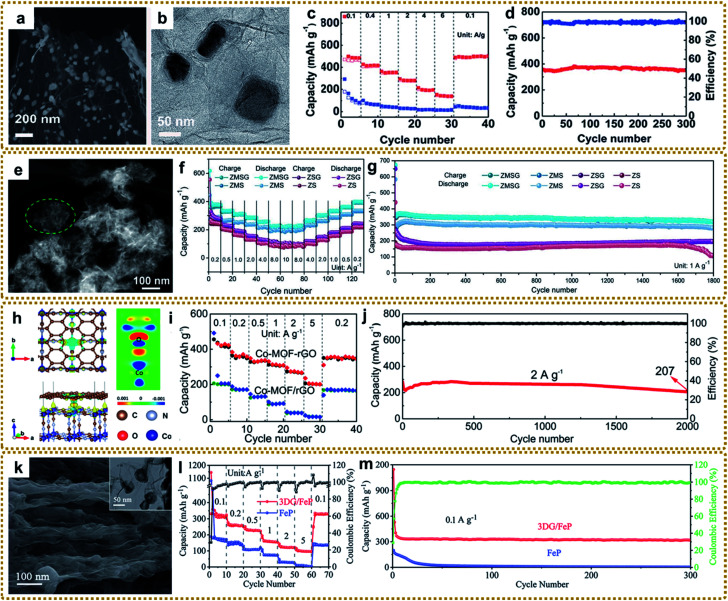
(a) SEM and (b) TEM images of core–shell 3DG/FeS@C. (c) Rate performance of 3DG/FeS@C and pure FeS. (d) Cycling performance of 3DG/FeS@C at 1 A g^−1^. Reproduced with permission from ref. 60. Copyright 2018, Royal Society of Chemistry. (e) HAADF-STEM image of the ZnSe⊂N–C@MoSe_2_/rGO hybrid. (f) Rate performance of different electrodes. (g) Cycling performance of different electrodes at 1.0 A g^−1^. Reproduced with permission from ref. 84. Copyright 2020, Wiley-VCH. (h) Top and side views of the charge density difference (CDD) of the Co-MOF–rGO hybrid. The yellow and blue regions represent positive (electron accumulation) and negative (electron depletion) values (in 0.001 e bohr^−3^) (left), respectively, and the two-dimensional slice of the CDD of the Co-MOF–rGO hybrid (right). (i) Rate capability of the Co-MOF–rGO hybrid and Co-MOF/rGO anodes. (j) Cycling performance of Co-MOF–rGO hybrid anodes at 2 A g^−1^ over 2000 cycles. Reproduced with permission from ref. 40. Copyright 2020, American Chemical Society. (k) SEM image of the side-view of the pressed 3DG/FeP film. The inset is the TEM image of 3DG/FeP. (l) Rate performance of 3DG/FeP and FeP. (m) Cycling performance of 3DG/FeP at 2 A g^−1^ over 2000 cycles. Reproduced with permission from ref. 112. Copyright 2020, Royal Society of Chemistry.

Morphology and structure engineering are effective strategies to boost the electrochemical properties of SIBs. The core–shell structure can buffer the volume change and prevent the active core material from pulverizing and aggregating during electrochemical processes.^111,145^ Our group fabricated an exquisite 3DG composite with a core–shell FeS@C encapsulated in 3DG by one-step thermal transformation of a 3DG/MOF composite, which exhibited excellent rate capacities of 363.3 and 152.5 mA h g^−1^ at 1 and 6 A g^−1^, and outstanding cycling stability with a capacity retention of 97.9% after 300 cycles at 1 A g^−1^, when directly used as a flexible anode for SIBs (Fig. 13a–d).^60^ In another study, Li *et al.* synthesized porous core–shell structured CoP@FeP microcubes interconnected by RGO through direct phosphorization of a GO wrapped core–shell Co(OH)_2_@PB nanocomposite.^170^ The well-designed structure showed high reversible capacity, and excellent rate capability and cycle life. A hierarchical hybrid structure with multicompositional features will bring about an unexpected synergistic effect for enhanced SIB performance. For example, Shi *et al.* synthesized a sandwich hierarchical architecture with ZnSe nanoparticles fastened in N-doped carbon polyhedra anchored onto graphene with the modification of MoSe_2_ nanosheets outside (ZnSe⊂N–C@MoSe_2_/rGO, ZMSG) *via* a self-template of MOFs and subsequent selenization strategy (Fig. 13e).^84^ The hybrid delivered a high rate capability of 224.4 mA h g^−1^ at 10 A g^−1^ (Fig. 13f) and extraordinary cycling performance that preserved 319.4 mA h g^−1^ after 1800 cycles at 1 A g^−1^, much higher than the performance of the single component (Fig. 13g). In addition, the performance of SIBs can be improved by constructing hollow structures and ultra-small nanocrystals.^63,72,150^

Compared to sodium, potassium possesses a lower reduction potential (K: −2.93 V and Na: −2.71 V *vs.* the reversible hydrogen electrode (RHE)), which allows potassium-ion batteries (PIBs) to have higher energy densities than SIBs. Besides, K^+^ exhibits a weaker solvation effect due to weaker Lewis acidity, ensuring its quicker kinetics in PIBs.^214,219^ However, the larger ionic radius (K: 1.38 Å and Na: 1.02 Å) will cause greater difficulty for K^+^ intercalation and a dramatic volume change during the discharge–charge process. Therefore, the electrode materials should be designed with large channels to promote the efficient insertion or extraction of K^+^. Due to the adjustable pore structure and relatively open channels as well as high specific surface area, MOFs are considered to be suitable for reversible K^+^ storage and transmission. Our group constructed a Co-MOF–rGO hybrid with Co-MOFs tightly encapsulated into rGO by growing Co-MOF nanocrystals on GO *via* synergistic coordination and electrostatic interactions and a subsequent annealing strategy.^40^ We found that there existed a chemical-bonded interface between Co-MOF nanocrystals and rGO (Fig. 13h), and the strong chemical interaction at the interface could substantially enhance the adsorption energy and ion transport kinetics of K^+^ within the Co-MOF nanocrystals compared to the physical mixture of Co-MOF and rGO. The resultant Co-MOF–rGO with strong interfacial chemical couplings showed superior rate capacities of 422 and 202 mA h g^−1^ at 1 and 5 A g^−1^, and outstanding long-term cycling performance with 74% capacity retention after 2000 cycles at 2 A g^−1^ (Fig. 13i and j). Besides, like LIBs and SIBs, some metal compound hybrids with distinguished structures derived from MOF/graphene composites (*e.g.*, hierarchically porous 3DG/FeP aerogel,^112^ RGO wrapped FeS_2_ hollow nanocages,^146^ graphene encapsulated Co_0.85_Se hollow cubes,^220^ and multicompositional ZnSe–FeSe_2_/RGO composites^88^) were explored as anode materials for PIBs. In particular, the flexible 3DG/FeP anode with hollow FeP nanospheres encapsulated within a 3D graphene skeleton delivered a high reversible capacity of 323 mA h g^−1^ at 0.1 A g^−1^ and ultrastable cycle life with a capacity retention of 97.6% at 2 A g^−1^ after 2000 cycles (Fig. 13k–m).^112^

### Lithium–sulfur batteries

Lithium–sulfur batteries (LSBs) are appealing as next-generation electrochemical energy storage devices because of their ultrahigh capacity (1675 mA h g^−1^) and theoretical energy density (2600 W h kg^−1^), and environmental benignity as well as the natural abundance of the sulfur cathodes.^34^ Unfortunately, LSBs are facing some severe challenges, which hinder their commercialization. For example, the insulating nature of sulfur (electrical conductivity: 5 × 10^−30^ S m^−1^ at 25 °C) leads to inferior electron transfer ability and poor coulombic efficiency. Besides, the large volume change of sulfur (∼80%) during cycling damages the integrity of the cathode structure. What's worse, the dissolution of intermediate polysulfides (Li_2_S_*n*_, 4 ≤ *n* ≤ 8) and their “shuttle effect” result in the loss of active materials. These drawbacks give rise to low capacity and poor cycling stability.^115,221^ To date, several approaches have been developed to address these issues, including the structural design of host materials, development of functional separators and new electrolytes, and modification of the lithium anode.^222^ In this context, MOF/graphene composites and their derivatives play an important role in improving the performance of LSBs.^223,224^

**Fig. 14:**
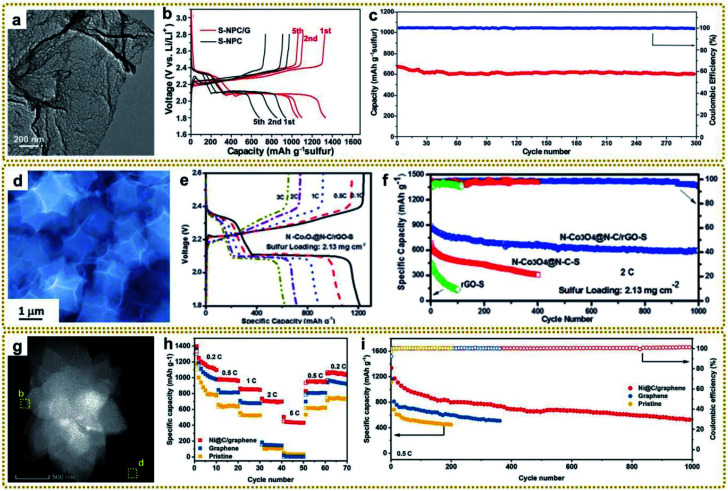
(a) TEM image of the NPC/G hybrid. (b) Rate performances of S-NPC/G and S-NPC cathodes under various current rates. (c) Cycling performance and coulombic efficiency of the S-NPC/G cathode at 1C. Reproduced with permission from ref. 115. Copyright 2018, Wiley-VCH. (d) TEM image of N–Co_3_O_4_@N–C/rGO–S. (e) Galvanostatic charge/discharge voltage profiles of the N–Co_3_O_4_@N–C/rGO–S cathode at step current densities from 0.1C to 3C. (f) Cycling performance test of the N–Co_3_O_4_@N–C/rGO–S, N–Co_3_O_4_@N–C/rGO–S and rGO–S electrodes at a 2C discharge rate and corresponding coulombic efficiency (sulfur loading: 2.13 mg cm^−2^). Reproduced with permission from ref. 232. Copyright 2018, Royal Society of Chemistry. (g) HAADF-STEM image of Ni@C/graphene. (h) Rate capabilities and (i) long-term cycle performance of the cells with Ni@C/graphene-modified, graphene-modified, and pristine separators at 0.5C (60% S). Sulfur mass loading: 2.0 mg cm^−2^. Reproduced with permission from ref. 234. Copyright 2020, Wiley-VCH.

Heteroatom doped carbon has been demonstrated to be a favorable host material due to its large surface area, abundant pore structure, and high electrical conductivity, as well as physical confinement and chemical adsorption of polysulfides. Chen *et al.* prepared a nitrogen-doped porous carbon anchored on graphene sheet (NPC/G) hybrid by thermal treatment of a ZIF/GO composite (Fig. 14a).^115^ In this architecture, the interconnected graphene could provide a highly conductive framework to facilitate rapid electron transport, and the abundant pore structure and doped N atoms could effectively trap polysulfides *via* both physical confinement and stronger chemisorption. Thanks to the multiple advantages, the NPC/G-based sulfur cathode (S-NPC/G) presented a high specific capacity of 1372 mA h g^−1^ and good cycling stability over 300 cycles (Fig. 14b and c). In a separate study, a 3D porous carbon framework composed of hollow carbon polyhedra coated with rGO was chosen as a host material for LSBs, which suppressed the “shuttle effect” during the charge/discharge cycles due to the unique carbon structure and N doping, leading to high discharge capacities and excellent cycling stabilities.^121^ To further trap the polysulfides, Tan *et al.* coated sulfur-filled rGO/ZIF-8(C) derived from GO/ZIF-8 with poly(3,4-ethylenedioxythiophene) (PEDOT) outside. The resultant composite cathode exhibited higher capacity and longer cycle life than that without PEDOT coating.^225^ Furthermore, Co,N-co-doped porous carbon hosts showed enhanced LSB performance.^226–229^ For example, Wang *et al.* fabricated a free-standing sulfur host consisting of Co,N-co-doped porous carbon nanocages on 3D rGO (Co/N–PCN@rGO) through pyrolyzation of ZIF-67@rGO followed by acid leaching.^226^ After being penetrated by sulfur, the obtained cathode manifested a high discharge capability and an extremely low capacity decay rate, which was better than that of most reported carbon–sulfur-based cathodes.

Polar metal compound (*e.g.*, oxide, sulfide, and phosphide) based sulfur hosts derived from MOF/graphene nanocomposites were extensively studied due to their strong adsorption of polysulfides on polar surfaces.^107,162,230–232^ For example, rGO wrapped mesoporous MoO_2_ microrods derived from Mo-MOFs were synthesized and used as the sulfur host in LSBs. The metallic MoO_2_/rGO composite not only enhanced the transport of electrons and Li^+^ diffusion, but also restricted the dissolution and shuttling of polysulfides. Moreover, the catalytic effects of MoO_2_ facilitate polysulfide conversion kinetics. Therefore, the S–MoO_2_/rGO cathode exhibited a high discharge capacity of 1145 mA h g^−1^ at 0.5C and ultralong cycle life over 3200 cycles at 3C.^230^ Particularly, Xu and co-workers confirmed that the doping of nitrogen into cobalt oxides could provide additional affinity sites and strengthen the binding energy for polysulfide absorption.^232^ As a result, the well-defined porous N–Co_3_O_4_@N–C/rGO synthesized by pyrolysis of ZIF-67 and subsequent rGO wrapping delivered a high reversible capacity of 1223 mA h g^−1^ at 0.2C and excellent cycling stability of 611 mA h g^−1^ retained at 2C after 1000 cycles (Fig. 14d–f). In addition, to increase the areal capacity, a higher sulfur loading is generally required. He *et al.* constructed a MOF-derived hollow Co_9_S_8_ array anchored onto a 3D graphene foam (Co_9_S_8_-3DGF) as a free-standing sulfur host for long-life LSBs. The Co_9_S_8_-3DGF/S cathode containing an ultrahigh sulfur loading of 10.4 mg cm^−2^ realized an incredibly high areal capacity of 10.9 mA h cm^−2^ at a rate of C/10 and still good cycling stability for 200 cycles.^107^

Apart from being used as host materials, MOFs/graphene and the derived nanocomposites have also been applied as functional separator modifiers to mitigate the problem of polysulfide crossover. In this way, polysulfides can be efficiently confined in the cathodic side. Bai *et al.* reported a well-designed MOF@GO ionic sieve as a separator for improved LSB performance.^233^ Due to the significantly smaller micropore size (approximately 9 Å) of the used HKUST-1 than the diameters of lithium polysulfides, the soluble polysulfides were efficiently blocked at the anode side while Li^+^ ions were selectively sieved. In this case, LSBs with the MOF-based separator achieved a low capacity decay rate of 0.019% per cycle over 1500 cycles. Besides, Yu and co-authors fabricated a modified separator with electrocatalytic activity by dispersing Ni-MOF-74/GO derived graphene-supported Ni nanoparticles with a carbon coating (Ni@C/graphene) on a commercial glass fiber membrane (Fig. 14g). DFT calculation and an electrochemical investigation indicated that the Ni@C/graphene-modified separator could regulate the catalytic conversion of polysulfides and served as sulfiphilic sites to suppress the shuttle effect in the whole Li–S redox process. Benefiting from these merits, the corresponding LSBs showed a much better rate capability and superior cycle performances (Fig. 14h and i).^234^

### Oxygen evolution and reduction reactions (OER and ORR)

The oxygen evolution reaction (OER) and oxygen reduction reaction (ORR) are two important parts of electrochemical energy conversion systems. The OER is a key electrochemical reaction involved in rechargeable metal–air batteries, fuel cells, and water electrolysis for oxygen and hydrogen generation with the mechanism of oxidation of water/hydroxyl ions on the surface of electrocatalysts and simultaneous evolution of oxygen gas. Meanwhile, the ORR is the reverse reaction of the OER, and is a critical process in metal–air batteries and fuel cells.^235,236^ The two reactions dominate the performance of these electrochemical energy conversion devices to a large extent. Unfortunately, the inherent reaction kinetics of the OER and ORR are sluggish due to the multiple proton-coupled electron transfer procedures. To date, noble metal-based catalysts are still highly efficient for these reactions. However, their scarcity, high cost, and poor stability undoubtedly limit the large-scale applications.^237^ Therefore, developing low-cost and noble metal-free catalysts with reliable performance is highly demanded. Recently, research on MOF/graphene-based materials has provided an opportunity to develop efficient electrocatalysts.

**Fig. 15:**
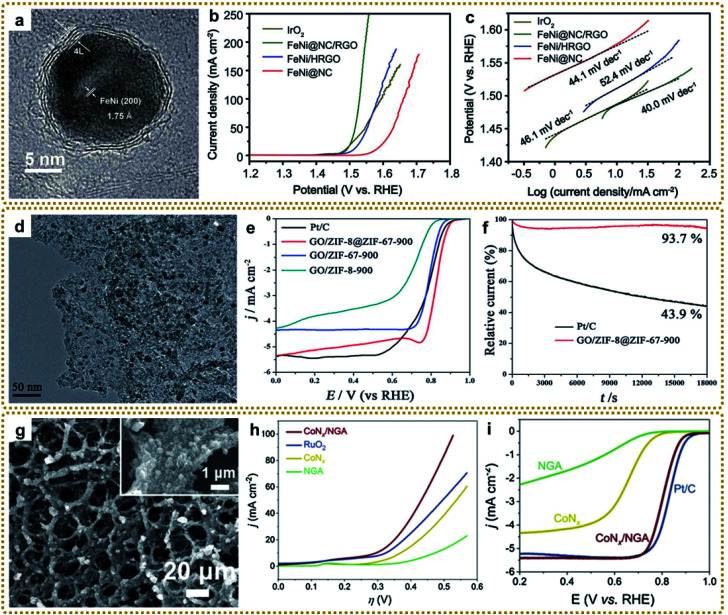
(a) HRTEM image of FeNi@NC/RGO. (b) IR-corrected polarization curves, and (c) Tafel curves of FeNi@NC/RGO, FeNi/HRGO, FeNi@NC and IrO_2_. Reproduced with permission from ref. 44. Copyright 2018, Royal Society of Chemistry. (d) TEM image of GO/ZIF-8@ZIF-67-900 prepared by carbonization of GO/ZIF-8@ZIF-67 at 900 °C followed by acid etching. (e) LSV curves of Pt/C, GO/ZIF-8@ZIF-67-900, GO/ZIF-67-900 and GO/ZIF-8-900 at 1600 rpm in O_2_-saturated KOH solution (0.1 M). (f) Current–time chronoamperometric response of Pt/C and GO/ZIF-8@ZIF-67-900 at 0.80 V (*vs.* the RHE) with a rotating speed of 1600 rpm in O_2_-saturated 0.1 M KOH solution. Reproduced with permission from ref. 116. Copyright 2017, Royal Society of Chemistry. (g) SEM images of CoN_*x*_/NGA. (h) LSV curves for CoN_*x*_, NGA, CoN_*x*_/NGA, and RuO_2_ with a scan rate of 5 mV s^−1^ in 1.0 M KOH solutions. (i) LSV curves at a rotational speed of 1600 rpm for CoN_*x*_, NGA, CoN_*x*_/NGA, and Pt/C with a scan rate of 5 mV s^−1^ in 0.1 M KOH solutions. Reproduced with permission from ref. 179. Copyright 2019, Elsevier.

MOF/graphene composites have been reported as direct active catalysts for the OER. Chen *et al.* developed a lamellar bimetallic MIL-53(FeNi) encapsulated in graphene aerogel-grafted Ni foam by an *in situ* solvothermal approach.^110^ The metal centers located in the edge-sharing octahedral MO_6_ layers in MIL-53 helped to optimize the absorption energies of the oxygen-containing intermediates. And the porous graphene aerogel could act as a conductive bridge to connect MIL-53 crystals with Ni foam. As a result, the catalyst showed excellent OER performances in alkaline media. In another study, a highly active Co_*x*_Ni_1−*x*_ MOF/3DG electrocatalyst was prepared by a similar method for the OER, which exhibited a high catalytic activity attributed to the synergistic effect of Co and Ni in MOFs and the 3D structure of graphene.^238^ Besides, various MOF/graphene-derived composites (*e.g.*, metal or alloys,^44,239^ oxides,^131,133,136,139^ sulfides,^153,154^ phosphides,^45,166–169,172^ nitrides,^180^*etc.*^157^) have been demonstrated as active OER electrocatalysts. For instance, our group developed an ultrafast microwave-assisted CVD-like method to convert a MOF/GO precursor into well-dispersed core@shell metal@NC nanocrystals with few-layer NC on RGO (M@NC/RGO) composite (Fig. 15a).^44^ The huge heat rapidly generated under microwave radiation enabled the CVD-like generation of M@NC nanocrystals. It was believed that N-doping in the graphene shell could increase the density of states (DOS) near the Fermi level and few-layer NC could facilitate electron transfer from the metal core to the graphene shell. Benefiting from these structural advantages, the obtained FeNi@NC/RGO displayed a low overpotential of 261 mV at 10 mA cm^−2^ in 1 M KOH and a small Tafel slope of 40 mV dec^−1^, which was superior to that of a commercial IrO_2_ catalyst (Fig. 15b and c).

The intrinsically unique structures provide infinite possibilities for the application of MOF/graphene-based materials in different electrocatalytic systems, including the ORR. Metal-free N-doped porous carbon/rGO composites derived from ZIFs/GO by high temperature carbonization have been extensively used as electrocatalysts for the ORR and presented comparable performance to commercial Pt/C, due to the synergistic effect between porous NC and graphene with regard to structure and composition.^113,114,119,240^ To further improve the ORR performance, embedding metal nanoparticles or single atoms into nitrogen-doped carbon (M–N–C, M = Fe, Co, *etc*.) is a good choice.^116,128,129,241–244^ On the one hand, the NC structure could play its original role to facilitate fast mass transport and electron transfer. On the other hand, the additional M–N_*x*_ active sites could promote oxygen adsorption and accelerate the ORR kinetics, thus leading to better ORR performance. For example, Wei *et al.* fabricated Co nanoparticles/N-doped porous carbon nanosheets through the pyrolysis of a sandwich-like GO/ZIF-8@ZIF-67 precursor at 900 °C, which was synthesized *via* a ZIF-8 seed-mediated deposition route (Fig. 15d).^116^ The obtained GO/ZIF-8@ZIF-67-900 catalyst delivered a high onset potential (∼0.93 V *vs.* RHE), which was a slightly higher than that of Pt/C but better than that of porous NC nanosheets prepared under the same conditions (Fig. 15e). Furthermore, it showed better durability than Pt/C under alkaline conditions (Fig. 15f). To maximize the atom-utilization efficiency and achieve abundant M–N_*x*_ active sites, porous Fe–N-doped graphene nanosheets with single-atom Fe-sites on the surface were prepared, which exhibited an outstanding ORR activity.^128^ Besides, many metal compounds derived from MOFs/graphene were also developed for high-performance ORR catalysts.^132,137,175,177^

In recent years, more efforts have been devoted to the development of efficient and stable bifunctional oxygen electrocatalysts due to their practicability. Up to now, various high-activity catalysts based on MOF/graphene composites, including M–N–C,^245,246^ bimetallic sulfides,^92,152^ metal nitrides,^179,181^ and metal oxides and carbides,^94,178^ showed great advantages and application potentials for the OER and ORR. In particular, cobalt-based nitride/graphene hybrids have attracted much attention because of their tailored electronic configuration of Co sites coordinated by N atoms and high inherent per-site catalytic activity. For example, a 3D nitrogen-doped graphene aerogel coupled with amorphous cobalt nitride (CoN_*x*_/NGA) was reported by Zou and co-authors (Fig. 15g).^179^ The hybrid with a highly open 3D hierarchical NGA structure offered a highway for electron transfer and shortened pathways for mass transportation. Moreover, the abundant active CoN_*x*_ and N_*x*_C sites stemming from amorphous CoN_*x*_ particles and NGA greatly boosted the electrochemical reactions. As a result, the integrated advantages guaranteed the high activity of CoN_*x*_/NGA toward both the OER and ORR, which is comparable with noble metal catalysts (Fig. 15f and g).

### CO_2_ reduction

**Fig. 16:**
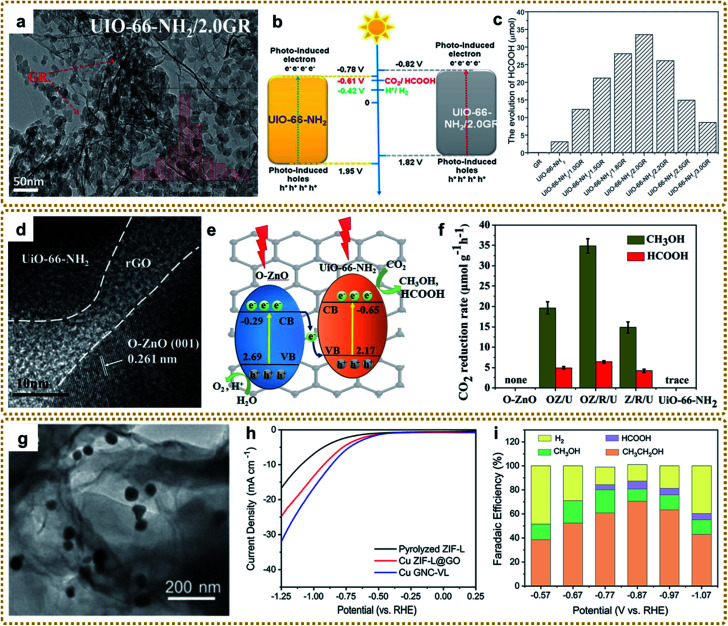
(a) TEM image of UIO-66-NH_2_/2.0GR synthesized by the MIS route. (b) Potentials of valence and conduction bands of UIO-66-NH_2_ and UIO-66-NH_2_/2.0GR. (c) The evolution of formic acid of various photocatalysts under visible-light (*λ* > 410 nm) irradiation. Reproduced with permission from ref. 248. Copyright 2018, Elsevier. (d) HRTEM image of OZ/R/U. (e) CO_2_ reduction rate of the as-prepared samples. (f) Schematic illustration of the charge transfer and separation in O–ZnO/rGO/UiO-66-NH_2_: Z-scheme mechanism. Reproduced with permission from ref. 251. Copyright 2019, American Chemical Society. (g) High-angle annular dark-field scanning transmission electron microscopy image of Cu GNC-VL. (h) LSV curves of pyrolyzed ZIF-L, Cu ZIF-L@GO, and Cu GNC-VL in CO_2_-saturated 0.5 M KHCO_3_ electrolyte with a scan rate of 10 mV s^−1^. (i) The FE of the Cu GNC-VL catalyst. Reproduced with permission from ref. 253. Copyright 2020, American Chemical Society.

Catalyzing CO_2_ transformations into valuable chemicals and fuels (*e.g.*, hydrocarbons, HCOOH, CH_3_OH, C_2_H_5_OH, CO, *etc.*) is considered to be a promising way to alleviate global warming and energy issues.^247^ Photocatalytic CO_2_ reduction is an attractive approach owing to its direct use of visible light or sunlight as the energy source. A strong capability for CO_2_ capture, abundant reaction active sites, high electron–hole separation properties, and wide optical absorption spectrum are imperative for high-efficiency photocatalysts. In this regard, MOF/graphene-based composites were reported to be efficient for photocatalytic CO_2_ reduction.^248–251^ Wang *et al.* constructed a small-sized UIO-66-NH_2_ nanocrystals/graphene (UIO-66-NH_2_/GR) structure with a high dispersion and strong junctions of UIO-66-NH_2_ on the surface of graphene *via* a microwave-assisted *in situ* growth and assembly route (Fig. 16a).^248^ The small-sized UIO-66-NH_2_ nanocrystals could provide more active surface for trapping CO_2_ and generating photogenerated electrons, and the strong junctions could effectively promote the photoelectron–hole separation. In addition, the UIO-66-NH_2_/2.0GR presented a more negative conduction band and lower band gap (Fig. 16b), which was beneficial for the enhanced ability of CO_2_ reduction and longer-wavelength light absorption. Finally, the UIO-66-NH_2_/2.0GR composite exhibited excellent activity and selectivity in the CO_2_ photo-reduction to HCOOH under visible-light (*λ* > 410 nm) irradiation (Fig. 16c). In order to further extend the visible light absorption range to the full spectrum for increased solar light utilization, Sadeghi *et al.* used porphyrins to modify a MOF and designed a graphene–porphyrin based MOF photocatalyst, which showed high efficiency and selectivity of visible light-driven CO_2_ to formate.^250^ In another case, a multi-component synergistic effect showed advantages for a high-activity photocatalyst. Meng and co-authors fabricated an oxygen-defective ZnO/rGO/UiO-66-NH_2_ (denoted as OZ/R/U) Z-scheme heterojunction *via* a solvothermal method (Fig. 16d).^251^ The CO_2_ reduction reactions over the OZ/R/U heterojunction under visible light followed the Z-scheme photocatalytic mechanism (Fig. 16e), which could effectively decrease the recombination rate of photogenerated charge carriers while maintaining their high redox capabilities simultaneously. Thus, the Z-scheme OZ/R/U heterojunction exhibited high photocatalytic activity with the yields of CH_3_OH and HCOOH reaching 34.85 and 6.40 μmol g^−1^ h^−1^, respectively (Fig. 16f).

The electrochemical CO_2_ reduction technology is also an effective route to convert CO_2_ to value-added products. However, it suffers from a large overpotential, low selectivity and catalytic efficiency, mainly due to the high thermodynamic stability of CO_2_ and possible multi-step reaction pathways during the proton-coupled electron transfer processes.^252^ Therefore, developing highly active, selective and stable catalysts is crucial for practical CO_2_ electroreduction. MOF/graphene-based materials provide an alternative solution for efficient CO_2_ electroreduction. Zhang *et al.* prepared Cu/Cu_2_O nanoparticles supported on vertically ZIF-L-coated nitrogen-doped graphene nanosheets (denoted as Cu GNC-VL) for electroreduction of CO_2_ to ethanol.^253^ The obtained Cu GNC-VL catalyst presented a 3D structure with high electronic conductivity and uniformly dispersed Cu/Cu_2_O active sites (Fig. 16g). Catalytic studies disclosed that Cu GNC-VL achieved a high current density of 10.4 mA cm^−2^ at −0.87 V *versus* RHE and excellent faradaic efficiency of 70.52% for ethanol production in 0.5 M KHCO_3_ solution (Fig. 16h and i), which was attributed to the synergy between the asymmetric chemical adsorption of CO_2_ on Cu(111) and favorable thermodynamics and kinetics of C–C coupling on Cu_2_O(111).

## Summary and outlook

MOFs have been extensively investigated as a new class of inorganic–organic hybrid materials in various applications due to their high specific surface area, tunable pore structures, adjustable composition and morphological features. Nevertheless, the intrinsically poor electrical conductivity and low stability restrict their practical applications, especially in the field of energy storage and conversion. Constructing MOF/graphene composites is an effective approach to alleviate these issues due to the synergic effects between MOFs and highly conductive graphene. More importantly, MOF/graphene-derived materials open up new paths toward their widespread applications. In this review, we first summarized comprehensively the latest methods of synthesizing MOF/graphene hybrids, including physical mixing, *in situ* growth, and excess metal-ion induced *in situ* growth, and the respective synthesis mechanism of each strategy involving the unique role of graphene was carefully discussed. Then we systematically discussed the formation mechanisms/methods of MOF/graphene-derived nanocomposites, including carbonaceous materials, single atom nanocomposites, and metal oxides, sulfides, phosphides, carbides, and nitrides with sophisticated structures, and their promising applications with a detailed analysis of the structure–property relationship in energy storage and conversion, such as SCs, LIBs, SIBs, PIBs, LSBs, OER, ORR, and CO_2_ reduction. The current issues of various energy storage and conversion devices and how to improve the performance by employing MOF/graphene-based materials were also presented. We hope that this review will help researchers in related fields to have a comprehensive understanding of the recent progress in MOF/graphene-based materials, and stimulate further development of new high-performance materials for energy storage and conversion in the future.

Though great advances have been achieved in this field so far, several key challenges as well as opportunities regarding high-performance MOF/graphene-based materials and future practical application should be considered. (i) The strength of interface interaction between MOFs and graphene can not only significantly affect the charge transfer but also has an important impact on the ion adsorption energy and diffusion kinetics, thus impacting the electrochemical performance. Therefore, exploring MOF/graphene-based materials with strong interface interaction is very necessary for enhanced electrochemical performance. Meanwhile, constructing MOF/graphene-derived materials with heterostructures or heteroatom doped active components can precisely regulate the local electronic configuration for better properties. (ii) Reducing the size of MOF nanocrystals and their derivatives on graphene to expose more active sites for efficient electrochemical reactions without aggregation is of profound importance for high-performance applications. It is essential to develop a new and controllable strategy to synthesize such ultrasmall nanomaterials. (iii) Although MOF/graphene-derived materials exhibit a porous structure, the arrangement of these pores is usually disordered, which will lead to relatively limited mass transport kinetics compared to the ordered pore structure. Therefore, a material morphology with a hierarchically ordered porous structure needs to be pursued. (iv) A profound understanding of the formation processes of MOF/graphene-derived nanocomposites and the structure evolution as well as the structure–property relationships is in high demand, where theoretical calculations and *in situ* techniques will play important roles in future studies. (v) The coulombic efficiency and volumetric performance should be further increased without sacrificing the capacity and rate through an elaborate balance of porosity, micro/nano-structures, *etc.* to meet the practical application. As a result, MOF/graphene-derived materials will definitely show a bright future toward energy storage and conversion.

## Author contributions

K. Wang and K. N. Hui contributed equally.

## Conflicts of interest

There are no conflicts to declare.
